# Neural Correlates of Decisions in Quasi-Realistic, Affective Social Interactions in Individuals With Violence-Related Socialization

**DOI:** 10.3389/fnbeh.2021.713311

**Published:** 2021-10-21

**Authors:** Juliana Wiechert, Axel Janzen, Anja Achtziger, Thorsten Fehr

**Affiliations:** ^1^Department of Neurology, University of Lübeck, Lübeck, Germany; ^2^Correctional and Rehabilitation Center (Justizvollzugsanstalt, JVA) Bremen, Bremen, Germany; ^3^Department of Political and Social Sciences, Zeppelin University, Friedrichshafen, Germany; ^4^Center for Advanced Imaging Bremen/Magdeburg, Bremen, Germany; ^5^Department of Neuropsychology, Center for Cognitive Sciences, University of Bremen, Bremen, Germany

**Keywords:** social decision making, violence-related socialization, fMRI, ecological validity, naturalistic decision making, self defense, reactive aggression, prosocial response

## Abstract

Appropriate social behavior in aggressive-provocative interactions is a prerequisite for a peaceful life. In previous research, the dysfunctions of the control of aggression were suggested to be modulated by enhanced bottom-up (sub-cortically driven) and reduced top-down (iso-cortical frontal) processing capability. In the present study, two groups of individuals with enhanced (EG) and normal (NG) experiences of violent acts during their socialization made binary behavioral decisions in quasi-realistic social interactions. These interactions were presented in short video clips taken from a first-person perspective. The video clips showed social interaction scenarios oriented on realistic everyday life situations. The behavioral data supported the distinct affective qualities of three categories of social interactions. These categories were labeled as aggressive–provocative, social–positive, and neutral–social interactions. Functional neuroimaging data showed extended activation patterns and higher signal intensity for the NG compared to the EG in the lateral inferior frontal brain regions for the aggressive provocative interactions. Furthermore, the peri-aqueductal gray (PAG) produced enhanced activations for the affective interaction scenarios (i.e., aggressive-provocative and social-positive) in both groups and as a trend with the medium effect size for the neutral interactions in the EG. As the individuals in the EG did not show open aggression during the functional MRIA (fMRI) investigation, we concluded that they applied individual self-control strategies to regulate their aggressive impulses immediately. These strategies appeared to be top-down regulated through the dorsal frontal brain areas. The predominant recruitment of the heteromodal cortices during the neural processing of complex social interactions pointed to the important role of the learning history of individuals and their socialization with differing levels of violent experiences as crucial modulators in convicts. Our data suggest that building or strengthening the association between prototypical social contexts (e.g., aggressive-provocative interactions) and appropriate behaviors as a response to it provides a promising approach to successfully re-socialize people with a delinquent history.

## Introduction

### What Is Real Violence and How Can We Examine Its Neural Correlates in the Human Brain Validly?

Reactive aggression or reactive violence is a term that refers to the behavior in response to an assault or any kind of provocation (Tedeschi and Quigley, 1996; Gendreau and Archer, 2005; Blair, 2010; Ferguson and Dyck, 2012). There are only a few studies that reported on brain activation patterns related to reactive aggression in humans (e.g., Pietrini et al., 2000; Krämer et al., 2007; Lotze et al., 2007; Fehr et al., 2014). However, most of these studies lack ecological validity. The reason is that their experimental set-ups were designed to meet the requirements of the neuroscientific method that was used to measure brain activity (e.g., the tube of a magnetic resonance tomography, MRT). Hence, behavior that could count as a realistic response to a social conflict could not be performed in neuroscience laboratories (Fehr, 2012; Ferguson and Dyck, 2012; Fehr et al., 2014). Furthermore, the putative reactive aggressive behaviors shown under the laboratory conditions can be interpreted at best as minor aggressions provoked, for instance, in games. A good example of this kind of research is experimental designs such as modifications of the “Taylor Aggression Paradigm (TAP)” (Taylor, 1967; Ferguson and Dyck, 2012). The TAP is a competitive reaction time game played between a participant and a confederate of the experimenter. The confederate acts as a no-real opponent (i.e., the participant does not know that the opponent is not real). If the (alleged) opponent is slower than the participant, the participant is allowed to punish the opponent with an electric shock. The participants chose the intensity of the electric shock themselves. The opponents punish the participant, increasing the intensity of the electric shock up to an inappropriate intensity. This inappropriate behavior of the opponent is supposed to provoke a “tit-for-tat” response in the participant. Or, put differently, the behavior of the opponent is expected to provoke a revenge-like response in the participant. While the TAP (Taylor, 1967) has been discussed to provide a satisfying construct validity (Giancola and Zeichner, 1995), it has been doubted whether experimental approaches based on gaming-like procedures can measure the entire range of realistic reactive aggression. This range varies from simple feelings of mild provocation to participating actively in extremely violent acts such as an aggressive confrontation with a knife and more (see Fehr, 2012; Fehr et al., 2014). The “tit-for-tat” idea realized in experimental set-ups like the TAP (Taylor, 1967) appears to be a type of aggression that resembles revenge or retaliation. This kind of aggression can be observed often in everyday life (e.g., to honk and counter-honk in case of cutting the right of way of someone). Yet, situations wherein a person faces a realistic threat that requires (self-) defense behavior as their life and health might be in acute danger are not represented validly in the game-like experimental set-ups used to measure the neural correlates of aggression (see Tedeschi and Quigley, 1996; Fehr, 2012; Ferguson and Dyck, 2012). For this reason, more research is needed to explore and describe the complex picture of reactive aggressive behaviors in humans on both the behavioral and neural levels (see Fehr, 2012; Ferguson and Dyck, 2012).

The fundamental dilemma in the studies on brain functions in humans is that it is based on small sample sizes. Thousands of relevant variables and consequently inferential statistical tests are computed for small samples of around 10, 20, 30, or a few more participants. Most research on complex mental processes in neuroscience is exploratory and is, at most, useful in generating some reasonable hypotheses. In the case that the researchers would consider testing their predictions with statistical tests, it would take thousands of participants in the functional neuroimaging domain to fulfill the requirements for meaningful statistical testing (cf., Lieberman and Cunningham, 2009). Additionally, the more complex a mental process is, the more interindividually different characteristics, such as individual mental strategy, traits, or individual learning history, should be considered as explanatory factors of aggression (e.g., Fehr, 2013; Zilles and Amunts, 2013). Consequently, the multifactorial designs and inappropriate over-correction of type I errors decrease the rate of false positives, but at the same time, they reduce the exploratory value of a study and risk an inappropriately high rate of false negatives (type 2 error), which can be seen as a major problem in the neurosciences (cf., Lieberman and Cunningham, 2009).

### Brain Physiological Studies on Different Kinds of Reactive Aggression

In line with Lotze et al. (2007), the functional neuroimaging community began to substantially contribute to the research on the neural correlates of reactive aggression in the human brain in 2007. After more than one decade of investigation on this important and still underinvestigated scientific field, there are only a few studies on this topic, with exploratory value at best (e.g., Ferguson and Dyck, 2012). We argue that there is still a lot of work to do, and this work needs to be inspired by a broad range of methodological [i.e., functional MRI (fMRI) and electroencephalogram (EEG)] and experimental [i.e., classic experimental set-ups as the TAP (Taylor, 1967) (see also Krämer et al., 2007; Lotze et al., 2007)] innovations such as the usage of (quasi-)realistic stimulations (e.g., Fehr et al., 2014) and other approaches (see Tedeschi and Quigley, 1996; Wiswede et al., 2011; Fehr, 2012; Ferguson and Dyck, 2012; Fehr et al., 2014).

Lotze et al. (2007) claimed to be the first scientists who used a TAP-like, interactive experimental set-up in a functional neuroimaging study on social reactive aggression in humans. As in almost all fMRI studies, these authors recruited a rather small number of participants (*n* = 16), which limits the informative value of the study to an exploratory approach. Despite the small sample size, the work by Lotze et al. (2007) provided evidence that the ventral medial prefrontal cortices (vmPFC) of less sensitive individuals were more activated than those of the rather insensitive participants. Note that the sample size and the power of correlations in this study were small. Further, the correlations reported by Lotze et al. (2007) were based on beta-estimates that can be misinterpreted as a measure of blood oxygenation level dependent (BOLD)-activation intensities. Despite these weaknesses, the data presented by Lotze et al. (2007) appeared to confirm the data and models on the brain-physiological top-down control of impulsive behaviors by the inferior frontal brain regions (cf., Brower and Price, 2001; Blair et al., 2007; Blair, 2010).

On the other hand, it is surprising that retaliation, which is a specific kind of aggression in the isocortical brain areas [i.e., dorsal medial prefrontal (dmPFC) and occipital brain regions], was found as a neural correlate. It could be expected that realistic reactive aggression responses also activate the subcortical brain regions such as the periaqueductal gray or at least any of the limbic brain structures related to the processing of emotions (Gregg and Siegel, 2001; Siegel and Victoroff, 2009; Blair, 2010; Fehr et al., 2014). As Lotze et al. implied, it might be that the observed neural correlates of the putative retaliation response only reflected certain kinds of cooperative interaction and/or competitive behavior in decision-making.

In the same year, 2007, Krämer et al. (2007) also published the data collected while participants completed a modified version of the TAP and they were confronted with similar problems as in Lotze et al. (2007). Krämer et al. (2007) analyzed the fMRI data of a small sample of 15 participants, including individuals of both sexes. Surprisingly, they did not find any fMRI activation pattern that appears to be mandatory as a neural response to reactive aggressive scenarios (e.g., the periaqueductal gray). In the same study by Krämer et al. (2007), experimental tasks based on the tit-for-tat logic generated an anterior cingulate and anterior insula activation. These findings were interpreted as indicators of the processing of negative emotions. The anterior insula activation was linked to the positron emission tomography (PET) activation in a study on self-generated emotions by Damasio et al. (2000). But the even more important subcortical activation foci (i.e., activations in brainstem and midbrain structures) reported by Damasio et al. (2000) were completely ignored by Krämer et al. (2007). It appears that the data of Krämer et al. reflected the neural correlates of the cognitive factors driving reactive aggressive behavior in terms of retaliation or revenge. As the experimental set-up in the research of Krämer et al. resembled a game-like approach strongly (see above), we argue that the aggression induced in this study was a mild kind of aggression, just like what a player in a computer game experiences (cf., Tedeschi and Quigley, 1996; Regenbogen et al., 2010; Fehr, 2012; Ferguson and Dyck, 2012). Thus, the title of the paper by Krämer et al. (2007), “The neural basis of reactive aggression” might be somewhat hasty and claims to explain a broad range of aggressive behavior, but is rather referring to a specific, mild kind of aggression. Despite these inflated claims, the study conducted by Krämer et al. (2007) made a substantial contribution to the understanding of the neural correlates of revenge like reactive aggressions in the human brain.

In EEG studies, subcortical activations can be recorded, but the deeper in the brain these activations are generated, the less accurate they can be localized. For this reason, event-related potentials (ERPs) can be analyzed to localize the isocortical neural correlates, or in other words, the brain activity generated by sources close to the surface of the head. Hence, ERPs help us to understand the neural correlates of perception and cognition that might be related to the processing of reactive aggression. Such processes could be the top-down impulse control and perception-action cycle representation (see Blair, 2010; Fehr et al., 2014). Several recent studies investigated these ideas in groups of individuals with and without violent socialization histories (e.g., Krämer et al., 2008; Wiswede et al., 2011).

For instance, Wiswede et al. (2011) identified an early ERP-positivity around 200 ms after the stimulus presentation in 10 individuals who tended to be violent. Further, the same authors observed the relative negativity of ERPs around 300 ms over the frontal brain sites in the retaliation phase of the TAP (Zilles and Amunts, 2013) in 10 individuals who were less prone to violence. These temporal, frontal effects were interpreted as a neural correlate of the approach tendency in individuals prone to violence and as a neural correlate of the inhibitory top-down control behavior in individuals with a weaker tendency to violence. Unfortunately, in this research by Wiswede et al. (2011), neither the source nor topographical analyses were run to know if dorsal (i.e., an activation over premotor sites as potential neural approach correlate) or ventral (inhibition) components were related to the ERPs described above. Our study used fMRI to receive more precise spatial information about the distinct recruitment of the different frontal brain areas in the individuals who experienced more or less violence during their socialization (see below).

In contrast to Krämer et al. (2008); Wiswede et al. (2011) found two periods of late negativity over the frontal brain sites in an EEG-study while the participants completed the TAP (Taylor, 1967). The participants were divided into two groups, namely, a group of 20 economy students with low levels of aggression and a group of 20 economy students with high levels of aggression. The level of aggression correlated positively with the refraining of retaliation decisions only in the students with high levels of aggression. The early positivity of ERPs in the individuals prone to violence in Wiswede et al. (2011; see above), was interpreted as a neural correlate of the approach tendency, and the late negativity of ERPs reported by Krämer et al. (2008) was interpreted as a neural correlate of the top-down impulse control. These seemingly contrary results might be due to the differences between the samples in both studies. The findings suggest considering individual aspects such as violence-related traits, learning history, and socialization of participants. We intended to contribute to this idea by investigating participants with and without a delinquent background.

The processing of a threat with a proximal relevance should activate the flight-freeze-fight-system (Blair, 2010) in both animals (Gregg and Siegel, 2001) and humans (Siegel and Victoroff, 2009; Blair, 2010; Fehr, 2012; Fehr et al., 2014). Fehr et al. (2014) demonstrated that this is the case when quasi-realistic social interactions with strong provocation and threat potentials are processed in middle European male university students between 20 and 25 years of age with normal socialization.

After adapting the TAP (Taylor, 1967) by adding the option to avoid a situation (i.e., the FOE, fight-or-escape version of the TAP), Buades-Rotger et al. (2017) also observed the involvement of the peri-aqueductal gray (PAG) in a considerable number of 37 females deciding to remain passive or to withdraw. As males are discussed to be more often involved in physical reactive aggression behaviors than females (e.g., Strüber et al., 2008; Strüber and Fehr, 2009), it is somewhat surprising that Buades-Rotger et al. (2017) decided to examine females instead of males in aggression. In addition, these authors investigated and discussed the flight and fight concept in a study running reaction time game. In this game, the participants were confronted with the potential threat of having to endure loud tones in an already every noisy scanner (i.e., during an fMRI recording). The idea that these threats are valid operationalizations of aggression is rather far-fetched as the fight-flight system is typically discussed as the one in charge whenever a threat endangers the health and life of an individual (cf., Tedeschi and Quigley, 1996; Fehr, 2012; Ferguson and Dyck, 2012). Additionally, the avoidance of a stimulus (e.g., unpleasant noise) is not fleeing or escaping from a threat. Similarly, accepting the challenge of being possibly confronted with negative stimuli like loud noise, even under provocative gaming conditions, is not a legitimate fight. We conclude that the concepts of flight and fight do not apply to the interpreted data presented by Buades-Rotger et al. (2017) due to the experimental FOE design. Further, the study of Buades-Rotger et al. (2017) does not distinguish the different kinds of emotional categories (cf., Fehr and Herrmann, 2015). Therefore, the reported findings can neither be interpreted as emotional nor pure arousal effects. Despite these problems, Buades-Rotger et al. (2017) observed the involvement of the PAG areas during the “punishment” decision phase when comparing the high vs. low provocation conditions. This finding, combined with amygdala activation, might lead to the conclusion that some of the findings reflect an arousal modulated processing of fear. Yet, what Buades-Rotger et al. (2017) called “fight-decisions” activated a complex network of the brain areas that were also reliably shown by Fehr et al. (2007, 2014) in an experimental set-up that did not even require the participants to make decisions. Fehr et al. (2014) linked their results to the concept of neurally elaborated perception-action-cycle systems of learned complex and stereotypical social behaviors.

We argue that it is time to distinguish between the involvement of the PAG and other brain regions by considering the kind of experimental approach that was followed in a study, the characteristics of the sample, and the types of reactive aggression that were investigated. The types of aggression could be, for example, hot-emotional, threat-related, defense-inducing, and bottom-up, in contrast with the types of aggression that are rather cold-emotional, cognitive top-down, retaliation, or minor tit-for-tat like competitive forms of reactive aggressive responses (cf., Ferguson and Dyck, 2012). More studies are needed to enlighten the problem from different perspectives. The present research is a pilot work supposed to contribute to the neuroscientific investigations on aggression by following an experimental approach that was previously shown to be reliable (Fehr et al., 2007, 2014; Strüber and Fehr, 2009; Fehr and Achtziger, 2021) on two well-selected and matched samples of individuals with distinct socializations of violence.

In summary, only a few studies are activating realistic perceptual, behavioral, and proximally relevant threat-scenarios in functional neuroimaging set-ups (e.g., Pietrini et al., 2000; Fehr et al., 2007, 2014; Denson et al., 2009; Fehr, 2012). For instance, Pietrini et al. (2000) provided evidence that the down-regulation of the orbitofrontal areas might be a substantial contribution to the processing of defense like reactive aggression in the human brain (cf., Blair, 2004). In their study, healthy participants were asked to imagine a realistic attack on their mother in a narrow elevator and to let their reactive impulses flow freely in their minds. It was assumed that the impulses of the participants referred to the performance of defensive behavior. Fehr et al. (2007, 2014) and Strüber and Fehr (2009) displayed short video clips filmed from a first-person perspective. These clips showed realistic social interactions with varying contents (i.e., social-positive, reactive-aggressive, defensive, and emotionally neutral interactions). It turned out that these distinct kinds of social interactions generated largely overlapping, but also unique brain activation patterns each. These activation patterns were associated with learned and neurally elaborated stereotypic perception-action networks, emotional evaluations, and flight-fight behavioral impulses.

Even though gaming-like experimental approaches added important contributions to the neural processes underlying aggression (see above), it is worth delving deeper into this topic. We decided to do this by presenting realistic interaction scenarios of different emotional qualities and acquired participants actively involved in these actions. In particular, our experimental material covered a broad range of violent interactions (see Fehr, 2012; Fehr et al., 2014).

### The Present Pilot Study and Working Hypotheses

What individuals think during an experiment cannot directly be inferred by the brain activation patterns, especially not in the case of complex cognitive or emotional processes (e.g., Poldrack, 2006; Fehr, 2013). Thus, two prerequisites must be fulfilled for the valid interpretation of the functional neural signature data: (1) Besides a baseline condition (in the present study, the neutral condition), there must be at least one additional emotional or cognitive task category (in the present study, the social positive interaction category) to ensure that the results are not only due to general attentional and/or arousal related processing (e.g., Fehr and Herrmann, 2015). (2) the stimulus materials and procedures should be as realistic as possible, and all the stimuli should be carefully evaluated by the participants through ratings on scales (cf., Fehr et al., 2014). Based on the literature we discussed in the introduction, we decided to use a further developed version of an experimental approach by Fehr et al. (2014). Our non-student sample consisted of 30 males wherein 15 participants belonged to the experimental group (EG) (13 were inmates at the local correctional and rehabilitation center, see below for details) and 15 participants belonged to the control group (NG) (Non-violence Group). All the participants were asked to make decisions in social interactions of the following kinds during an fMRI recording: aggressive-provocative threatening interactions, social-positive (i.e., friendly) interactions, and neutral social scenarios (see below). The following hypotheses were proposed:

1. As a prerequisite for the valid interpretation of the brain physiological data, we measured the experience of the participants with violence during their socialization. We claimed that the EG participants would report more experiences with violent behaviors in their socialization and show higher levels of personality traits linked to aggression than the NG participants.
2. A further prerequisite for the valid interpretation of the brain physiological data is the evaluation of the video clips displaying the social interactions mentioned above by the participants after the main experiment (see also Fehr et al., 2014). In particular, the aggressive-provocative scenarios should result in higher scores in the fear and anger ratings (Ramirez and Andreu, 2006). Further, the EG participants should rate the aggressive-provocative scenarios as more familiar compared to the NG participants.
3. As one of the most important effects of aggressive-provocative contrasted to neutral scenarios on psychophysiological responses, we expected the involvement of the midbrain structures, such as the PAG, in all participants (Gregg and Siegel, 2001; Siegel and Victoroff, 2009; Blair, 2010; Fehr et al., 2014).
4. Following the idea that individuals whose socialization were shaped by experiencing high levels of violence lack an executive top-down control of behavior (e.g., Pietrini et al., 2000; Brower and Price, 2001; Blair, 2004), we expected for the contrast between the aggressive-provocative and neutral scenarios to be a larger extension of the middle and/or inferior frontal brain activation clusters (descriptively) and potentially a higher percent of signal change values for the NG as compared with the EG participants. This will be statistically tested for the jointly activated frontal brain areas.

## Experimental Procedures

### Participants

Thirty healthy male volunteers participated. Fifteen of them were members of the EG (age: 32.6 ± 10.3; education: 13.2 ± 2.4 years). The 15 members of the NG were matched with the EG members, considering their age and education. The participants of the NG had normal socialization and none of them reported a socialization background with high levels of violence (age: 31.4 ± 9.7; education: 13.2 ± 2 years). Thirteen of the participants in the EG were members of the open regime, and two of them were members of the closed regime of the local correctional and rehabilitation center. All the participants were right-handed according to the Edinburgh Handedness Inventory (Oldfield, 1971), and did not report any history of psychiatric or neurological illness, abnormal vision, regular drug use, or current medication with psychotropic side effects.

All participants were paid €15 for their participation. The EG participants additionally got a prolonged prison leave on the day of the data collection. All the participants were familiarized with the stimulus presentation, were informed about the entire procedure, and consented to participate in written form. The experimental set-up was designed according to the Code of Ethics of the World Medical Association (Declaration of Helsinki, published in the British Medical Journal, July 18, 1964), and the study protocol was approved by the ethics committee of the home university of the corresponding author.

### Design and Post-experimental Stimulus Evaluation

The participants watched 120 video clips ranging from 2.5 to 4 s (visual angle below 4°). These stimuli were a modified sub-sample of the video-clip inventory by Fehr et al. (2007, 2014) developed for the investigation of the neural correlates of social interactions of different affective qualities. We repeatedly presented 20 neutral (the interaction partner showed neither an aggressive-provocative nor social-positive behavior toward the spectator), 20 aggressive-provocative (the interaction partner attacked or provoked the spectator), and 20 social-positive (the interaction partner showed friendly behavior) scenarios. After displaying a video clip, a still image of the last video frame was shown. This image was the cue for the participant to decide whether to actively get involved in the interaction [i.e., to show approach behavior; right index finger pressed a button on magnetic resonance (MR)-compatible mouse] or to withdraw from the interaction (i.e., to show withdrawal behavior; this decision was made by pressing a button on an MR-compatible mouse with the right middle finger). After the decision, the video clip continued, revealing the consequences of the decision of the participant. The consequences were the following:

1. In neutral interactions, the affectively neutral-approach behavior (acting in accordance with the displayed course of action) or withdrawal behavior (e.g., stepping back) was shown.
2. In aggressive-provocative interactions, a reactive aggressive action was executed after an approach decision (e.g., pushing or hitting an assailant), while a withdrawal action was performed after a withdrawal decision of a participant (e.g., stepping back).
3. In social-positive interactions, prosocial behavior was shown in case a participant decided to approach the interaction (e.g., shaking hands or waving back to someone). In case a participant decided to withdraw from the interaction, withdrawal behavior was displayed in the video clip (e.g., stepping back).

**Figure 1:**
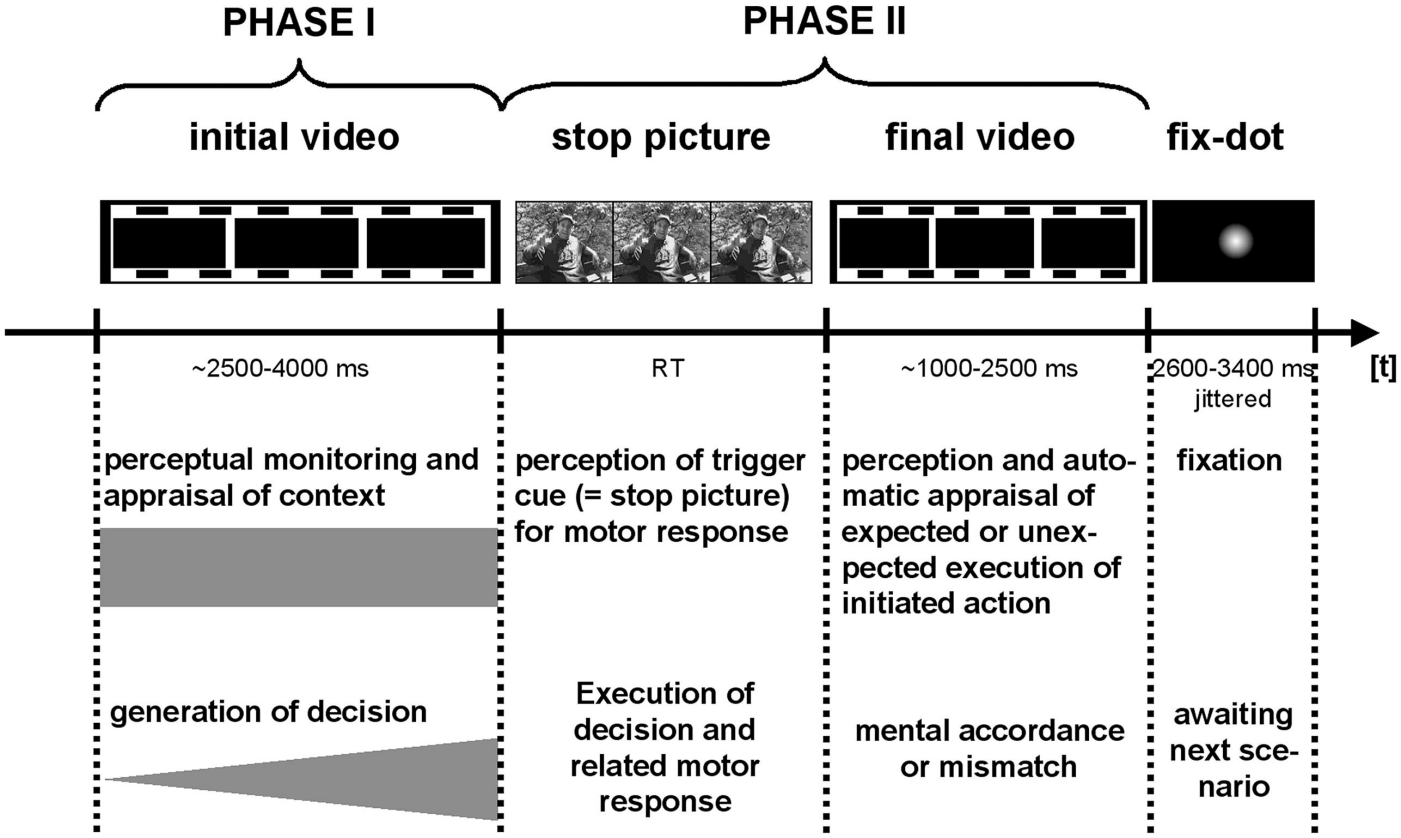
Trials elements and timing: Functional imaging data were separately modeled for PHASE I and PHASE II of the interaction scenarios, and the fixation period between trials.

The second part of the video clips (i.e., the part in that the consequences of the decision were presented) complemented the first part in a way that each video clip took 5 s. Hence, we manipulated a 3 s initial phase of a video clip that was followed by a 2 s phase that started after the participants decided to approach or withdraw the scenario by pressing a button (see above). Each video clip was displayed twice in the experiment, resulting in a total of 120 video-clip presentations and 120 decisions. All the video clips were filmed from a first-person perspective, ensuring the strong involvement of the observer in the highly realistic social interactions. Between each trial, a fixation dot was presented pseudo-randomly jittered between 2,600 and 3,400 ms (for the illustration of a trial see Figure 1).

For the fMRI sessions, two behavioral parameters were computed for each interaction category and participant. (1) The preference index (PI) (ranging from 0 to 1) representing the ratio of trials in which the participant chooses an approach behavior (reaching from 0 = *participant never decided to approach* to 1 = *participant always choose to approach*) within a specific interaction category. (2) The uncertainty index (UI) (ranging from 0 to 1) representing the ratio of the inconsistent decisions on the same video clip. Note that all the video clips were presented twice during the MRI scanning session. A score of 1 indicated a perfect inconsistency between the decisions on the same video clips. For instance, a participant decided to approach when he watched a certain scenario for the first time, and the second time he watched the same scenario, he chose to withdraw. In this example, his decision would have been inconsistent for this interaction. A score of 0 on the UI indicated that a participant made perfectly consistent decisions on all interactions.

The video clips were presented in a pseudo-randomized, non-stationary probabilistic sequence (Friston, 2000). This procedure was chosen because the emotionally laden stimuli typically show effects for longer than 30 s (Garrett and Maddock, 2001). The pseudo-randomized, non-stationary probabilistic sequencing of the stimuli provided a compromise between a block design (ideal for improving the signal-to-noise ratio) and an event-related design. The latter is perfect for psychological research because in this design each trial was processed without the expectancies cued by the emotions activated in the preceding trial (cf., Fehr et al., 2014).

After the MRI scanning session, all participants rated the *initial phase* of each video clip on an 11-point rating scale (ranging between 0 and 10; 0 = *no arousal/not familiar/not unambiguous*, 10 = *maximal arousing/familiar/unambiguous*) with AROUSAL and FAMILIARITY with the context shown in a video clip, and UNAMBIGUITY (i.e., how clear the presented scenario was for them). The participants also categorized the initial phase of each video clip regarding the emotion it evoked (EMOTIONAL CATEGORIZATION) into one of the following four categories: Fear or panic, anger or rage, friendliness, or neutral mood. Moreover, the participants categorized their BEHAVIORAL TENDENCY triggered by the initial phase of each video clip. That is, the participants indicated if they would approach the interaction, withdraw from it, or if they would remain passive in this situation.

Then, all the participants completed the following questionnaires: (1) the FAF (“Fragebogen zur Erfassung von Aggressivitätsfaktoren”; Engl.: “Questionnaire for the assessment of aggression factors/traits”; Hampel and Selg, 1975), which asks for self-ratings on the sub-dimensions of spontaneous (pro-active) aggression, reactive aggression, impulsiveness, auto-aggression, and inhibition, (2) the German version (Von Collani and Werner, 2005) of the Aggression Questionnaire (AQ) by Buss and Perry (1992) including the physical and verbal aggression, anger, and mistrust dimensions (3) the State-Trait Anger Expression Inventory (STAXI) (Spielberger, 1988; Schwenkmezger et al., 1992) measuring the trait anger, state anger, anger expression, and anger control, (4) the Six-Factor-Test (SFT) (“Sechs-Faktoren-Test,” a German personality inventory; Von Zerrsen, 1994) including the dimensions of extraversion, neuroticism, conscientiousness, aggression, openness to experiences, and religiosity, and finally (5) the Mehrfach-Wahl-Wortschatz-Test-B (MWT-B) (Lehrl, 2005), a standardized measure of verbal intelligence. The evaluations and categorizations of the video clips were run on a computer, and the questionnaires and verbal intelligence tests were completed as paper and pencil tests.

### Imaging Data Acquisition and Analyses

#### Data Acquisition, Pre-processing, and Regressor-Configuration

Functional and structural MRI data were obtained using a 3-T SIEMENS Magnetom Allegra® system (Siemens, Erlangen, Germany) equipped with a standard quadrature head coil. The changes in the BOLD T2^*^-weighted MR signal were measured using a gradient echo-planar imaging (EPI) sequence (44 3-mm thick axial (AC-PC) slices in interleaved acquisition order with a whole-brain coverage; FOV = 192 × 192 mm, 64 × 64 matrices, TR = 2500 ms, TE = 30 ms, flip angle = 90°). 450 ± 8.7 volumes were obtained during one complete run. Structural MRI data were collected after the functional scanning runs [magnetization prepared rage (MPRAGE)]; 160 slices, slice thickness of 1 mm, FOV = 256 × 256 mm; matrix: 256 × 256, TI = 900 ms, TR = 2,300 ms, TE = 4.38 ms; resulting in 1 mm3 of voxels with the same orientation).

Image analysis was performed using the Statistical Parametric Mapping software package (SPM5) (Welcome Department of Cognitive Neurology, London, UK) on a Matlab 6.5 platform (The MathWorks, Natick, MA, USA). For each session and participant, the images were slice-time corrected, realigned to the first image (motion-correction), and unwarped to reduce the potential effects of temporal, non-linear, magnetic field inhomogeneities, normalized to the Montreal Neurological Institute template (MNI) (Collins, 1994), and smoothed using an isotropic Gaussian kernel (full width half maximum = 8 mm) before further analysis. The global effects (grand mean scaling over all the volumes) were removed from the functional MRI data, and a high-pass filter (256 s) was applied to remove the low-frequency signal drifts.

All the video clips were split into an initial (PHASE I, between 2.5 and 4 s) and a final phase (PHASE II). Note that PHASE II took between 1 and 2.5 s depending on how long PHASE I lasted. Each trial was separately modeled for PHASE I and PHASE II and separately for the social-positive, aggressive-provocative, and neutral-interactions by a standard canonical hemodynamic response function. The neural processing of the fixation-dot displayed between each trial was also modeled separately (see Figure 1 for a detailed description of the trial structure).

As arousal was assumed to be modulated by the emotions generated by the interaction, the post-experimental ratings of the video clips (Phase I) were included as a regressor in the design matrices in the first-level analyses. The decision itself was also included as a dummy (0 = withdraw; 1 = approach) in the first-level analyses. As motion effects were critically discussed to potentially affect the functional imaging data (e.g., Lund et al., 2005), we included motion parameters (three volume-related translations and three rotation estimates for each volume) as regressors in the individual first-level design matrices.

#### Contrasting and Statistical Processing of Functional Imaging Data

The pre-processed data sets were analyzed by calculating a *t*-statistic for the pre-determined category effects at each voxel for each participant, producing a statistical image for different contrasts, including the stimulus conditions, namely, neutral (PHASE I: n1; PHASE II: n2), aggressive-provocative (PHASE I: a1; PHASE II: a2), and social-positive interactions (PHASE I: p1; PHASE II: p2).

Second-level random-effects analyses (Holmes and Friston, 1998) were performed on the individual contrast images to identify the main task effects, utilizing one-sample *t*-tests. A statistical threshold of *p* < 0.05 [false discovery rate (FDR) corrected, k ≥ 5 voxel cluster size] was applied to identify the significant activation clusters for the contrasting of aggressive-provocative (a) vs. neutral (n), and social-positive (p) vs. neutral (n) separately for each of the PHASEs I and II, and separately for both groups (EG and NG). The activation patterns common for both groups in PHASE I in the a vs. n interaction conditions were investigated through conjunction analyses (*p* < 0.05; FDR-corrected, k≥5 [cmp., Friston et al., 2005]). The MNI-coordinates of the peak activations were converted from the SPM5 output into Talairach space with a transformation algorithm, and a reference template based on the Talairach-atlas (Talairach and Tournoux, 1988) was used to determine the respective anatomical regions.

For exploratory purposes and as a basis for hypotheses building in future studies and for potential meta-analyses, we provided additional interaction analyses between the groups considering the subcortical and/or limbic, and inferior frontal brain areas (see Appendix 1 in the Supplementary Material for details).

#### Region of Interest (ROI) Analyses

There were four ROIs extracted for the activation clusters in the left and right inferior frontal gyri (lIFG and rIFG), the anterior thalamus (aTH) and PAG. These clusters were based on an inspection of activation clusters guided by our hypotheses (see above). The clusters were revealed by contrasts calculated separately for the two groups of participants (EG and NG). The percent signal change values were extracted by applying the software package Marsbar (Version 4.2; Brett et al., 2002).

## Results

### Behavioral Data

#### Inventory Test-Scores

Three sub-scales of the FAF (aggression inventory; Hampel and Selg, 1975) (z-values: spontaneous aggression [EG:0.1 ± 1; NG: −0.7 ± 0.8; *t* = 2.5, *p* = 0.02], impulsiveness [EG:0.5 ± 1.4; NG: −0.5 ± 0.8; *t* = 2.4, *p* = 0.02], and auto-aggression [EG:0.9 ± 0.8; NG: −0.2 ± 1.1; *t* = 3, *p* = 0.005]) showed higher scores for the EG compared to the NG. Further, the sum-score of the FAF indicated a trend toward lower levels of (trait) aggression in the NG (z-values: EG: 0 ± 1.4; NG: −0.9 ± 0.9; *t* = 1.9; *p* = 0.06; *d* = 0.73). For auto-aggression, the EG participants showed significantly higher scores compared with the norm-table (0.9 ± 0.8; *t* = 4.2, *p* = 0.0009). The NG participants, however, showed scores that were below the normal standards for the sub-scales: spontaneous (pro-active) aggression (−0.7 ± 0.8; *t* = −3.6, *p* = 0.003), reactive aggression (−0.8 ± 1.0; *t* = −3.1, *p* = 0.008), impulsiveness (−0.5 ± 0.8; *t* = −2.8, *p* = 0.02), and consequently, the summarized aggression score (−0.9 ± 0.9; *t* = −3.8, *p* = 0.002).

For the AQ (Von Collani and Werner, 2005), the participants in the EG rated themselves as higher in anger (EG: 2.6 ± 0.5; NG: 1.9 ± 0.5; *t* = 4.1, *p* = 0.0003), verbal aggression (EG: 2.7 ± 0.4; NG: 2.2 ± 0.5; *t* = 3.2, *p* = 0.004), mistrust (EG: 2.4 ± 0.6; NG: 2 ± 0.4; *t* = 2.4, *p* = 0.03), and physical aggression (EG: 2.6 ± 0.9; NG: 1.7 ± 0.4; *t* = 3.7, *p* = 0.0009) than the NG participants. The MWT-B (Lehrl, 2005) yielded higher verbal intelligence scores for the NG participants compared with the EG participants (*t* = 3.6, *p* = 0.001), means of both participant-groups (NG and EG) ranged between lower and average verbal IQ-levels (NG: 100.5 ± 9.2, EG: 91.8 ± 6.5; standardized average range: 91 to 109). The SFT-personality scores (Von Zerrsen, 1994) indicated a higher religiosity level for the EG members compared with the NG members (EG: 1.3 ± 0.8; NG:0.6 ± 0.5; *t* = 2.7, *p* = 0.01).

#### Post-experimental Evaluations of the Video Clips (PHASE I)

**Figure 2:**
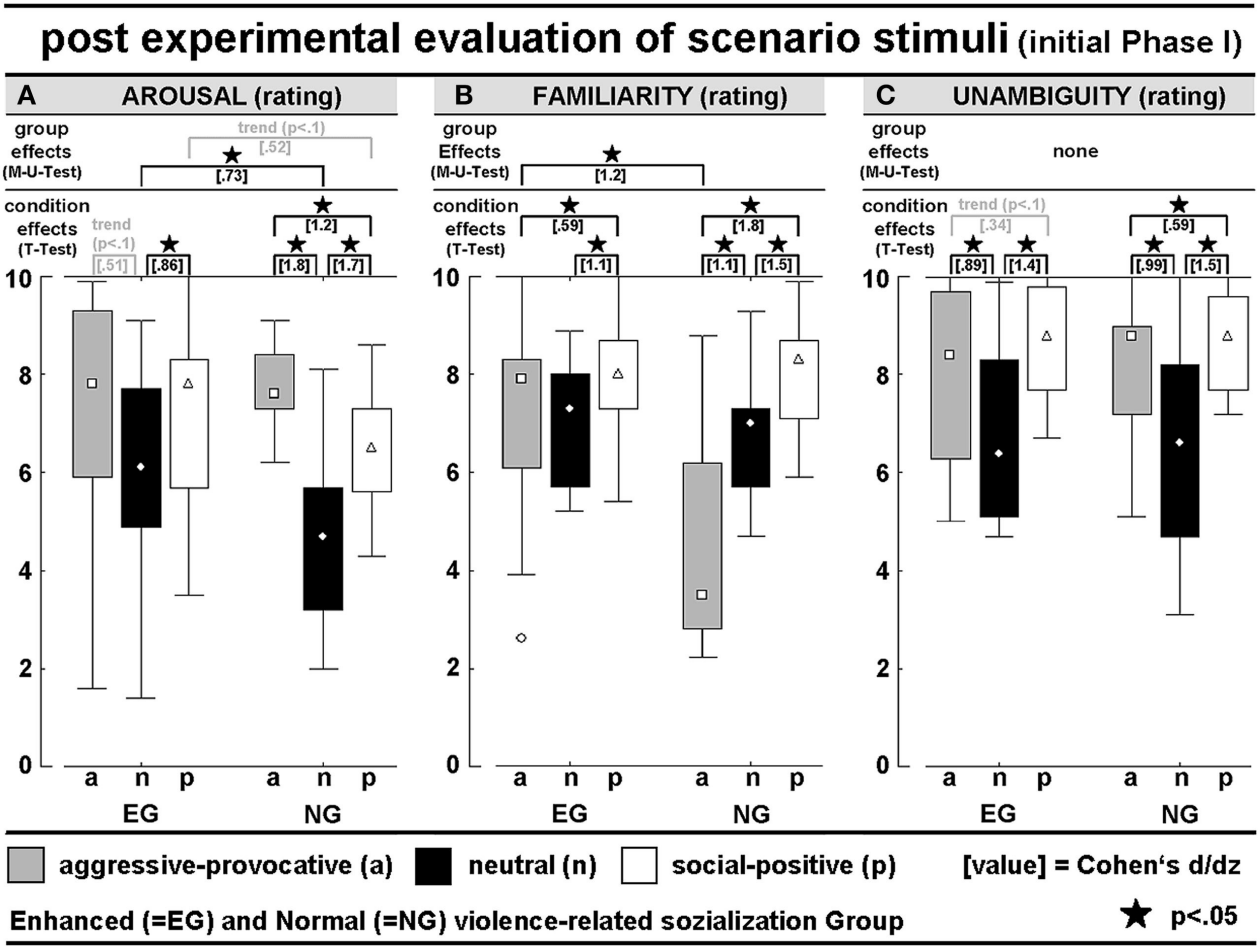
*Post hoc* evaluation of PHASE I of the video clips concerning **(A)** interaction-related arousal, **(B)** interaction-related familiarity rating, and **(C)** interaction-related unambiguity rating; all scales had 11 levels (0–10); see Table 1 for detailed numerical information.

AROUSAL-ratings: A mixed-model ANOVA (within the factor STIMULUS-CONDITION (SC) and between the factor GROUP) revealed the interaction-effect of GROUP × SC [*F*_(2, 56)_ = 3.3, *p* = 0.04; Greenhouse-Geisser (GG)-adjusted: *p* = *0.0*6], and the significant main effect of SC [*F*_(2, 56)_ = 20.2, *p* < 0.001; GG: *p* < 0.00001]. *Post hoc t*-tests revealed higher arousal scores for the aggressive-provocative and social-positive interactions compared with neutral interactions in the EG (aggressive-provocative vs. neutral: *t* = *2, p* = 0.07; social-positive vs. neutral: *t* = 3.3, *p* = 0.005). For the NG, the *t*-tests revealed higher arousal scores for the aggressive-provocative interactions compared with both social-positive and neutral interactions (*t* = 6.8, *p* < 0.00001, and *t* = 4.6, *p* = 0.0004), and social-positive compared with neutral interactions (*t* = 6.6, *p* = 0.00001). The EG-participants showed higher arousal scores than the NG-participants for the neutral [non-parametric Mann-Whitney-U(M-U)-test: Z = 2.0, *p* = 0.04879] and as a statistical trend for social-positive interactions (M-U-test: *Z* = *1.9, p* = *0.06*). The results are illustrated in Figure 2A.

FAMILIARITY-ratings: A mixed-model ANOVA (within the factor SC and between the factor GROUP) revealed the significant interaction of GROUP × SC [*F*_(2, 56)_ = 12.1, *p* = 0.00004; GG: *p* = 0.0004], and a main effect for SC (*F*_(2, 56)_ = 29.6, *p* < 0.00001; GG: *p* < 0.00001]. In the EG, the *post hoc t*-tests demonstrated higher familiarity scores for the social-positive interactions compared with both aggressive-provocative and neutral interactions (*t* = 2.3*, p* = 0.04 and *t* = 4.2, *p* = 0.0008). In the NG, the *post hoc* t-tests also showed higher familiarity scores for the social-positive interactions compared with both aggressive-provocative and neutral interactions (*t* = 6.9*, p* < 0.00001 and *t* = 5.7, *p* = 0.00005), and for the aggressive-provocative compared with the neutral interactions (*t* = 4.2, *p* = 0.00008). Moreover, the EG-participants revealed higher familiarity scores than the NG-participants concerning the familiarity with aggressive-provocative interactions (M-U-test: *Z* = 2.8, *p* = 0.006). The results are illustrated in Figure 2B.

UNAMBIGUITY-ratings: An ANOVA (within the factor SC and between the factor GROUP) found the significant main effect of SC [*F*_(2, 56)_ = 35.6, *p* < 0.00001; GG: *p* < 0.00001]. In the EG, the exploratory *post hoc t*-tests revealed higher unambiguity scores for social-positive interactions compared with both aggressive-provocative and neutral interactions (t = 2.1, *p* = 0.05 and *t* = 5.5, *p* = 0.00007), and higher unambiguity scores for aggressive-provocative compared with neutral interactions (*t* = 3.5*, p* = 0.004). In the NG, social-positive interactions produced higher unambiguity scores compared to both aggressive-provocative and neutral interactions (*t* = 2.3*, p* = 0.04 and *t* = 5.6, *p* = 0.00006), and aggressive-provocative interactions were rated higher on unambiguity than neutral interactions (t = 3.8*, p* = 0.002). The results are illustrated in Figure 2C.

BEHAVIORAL TENDENCY (BT)-ratings: An ANOVA (within the factors SC and BT, between the factor GROUP) resulted in a significant BT x SC interaction [*F*_(4, 112)_ = 11.5, *p* < 0.00001; GG: *p* = 0.00003], and the significant main effects for BT [*F*_(2, 56)_ = 15.5, *p* < 0.00001; GG-adjusted: *p* = 0.00002] and SC [*F*_(2, 56)_ = 54.9, *p* < 0.00001; GG-adjusted: *p* = 0.00002]. The pooled group statistics (i.e., including both the EG and NG-participants) indicated a general preference for reactive-aggressive approach behaviors in aggressive-provocative situations (approach vs. staying passively: *t* = 4.4, *p* = 0.0001; approach vs. withdrawal: *t* = 2.0, *p* = 0.06, as a statistical trend), followed by withdrawal (withdrawal vs. staying passively: *t* = 2.5, *p* = 0.02). Furthermore, as a response to the presentation of neutral interactions, the participants chose to stay passive or, as indicated by the statistical trend, in approaching the situation (staying passively vs. withdrawal: *t* = 2.3, *p* = 0.03; approach vs. withdrawal: *t* = 1.7, *p* = 0.0965). Additionally, the social-positive interactions predominantly led to approach behaviors (approach vs. remaining passive: *t* = 5.2, *p* = 0.00002; approach vs. withdrawal: *t* = 8.5, *p* < 0.00001) followed by remaining passive (remaining passive vs. withdrawal: *t* = 2.8, *p* = 0.01).

**Figure 3:**
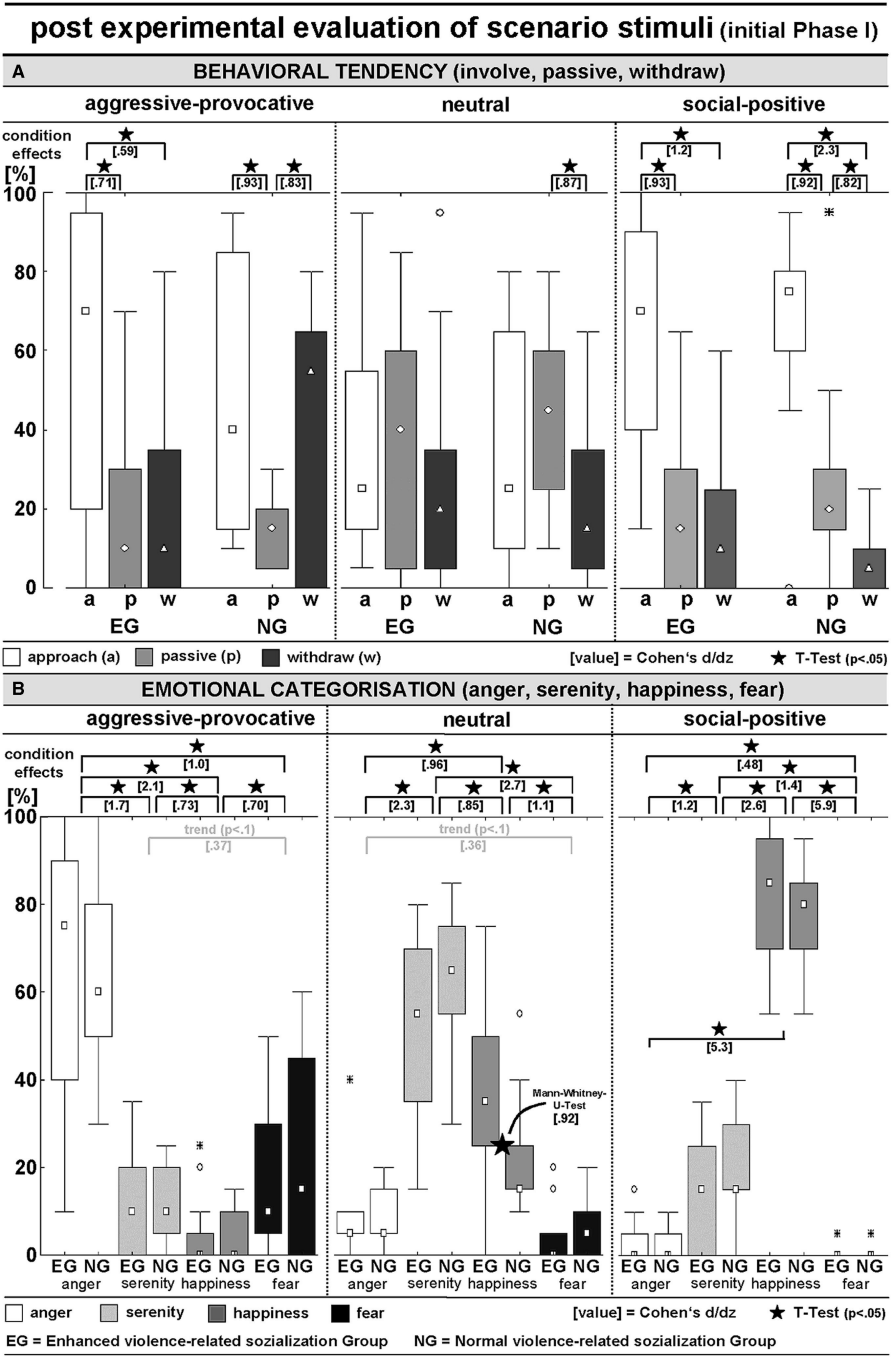
*Post hoc* evaluation of PHASE I of the video clips concerning **(A)** interaction-related behavioral tendency (approach, behave passively, or withdraw) and **(B)** interaction-related emotional appraisal (scenarios induced anger, serenity, happiness, or fear); see Table 1 for detailed numerical information.

The behavioral tendencies were characterized by heteroscedasticity and specificities in the skewness between the two groups of participants and the stimulus conditions (i.e., categories of the interactions; see Figure 3A). This potentially lowered the probability for consistent group effects and highlighted the potential of our stimulus inventory (i.e., the video clips) to explore individual differences in responding to highly specific kinds of social interactions. Thus, the group-related illustrations of the distributions of behavioral tendencies in Figure 3A should be cautiously interpreted but kept in mind as being of high interest.

EMOTIONAL CATEGORIZATION (EC): An ANOVA (within the factors SC and EC, between the factor GROUP) revealed a significant EC x SC interaction [*F*_(6, 168)_ = 175.3, *p* < 0.00001; GG-adjusted: *p* < 0.00001] and a main effect of EC [*F*_(3, 84)_ = 38.6, *p* < 0.00001; GG-adjusted: *p* < 0.00001]. The aggressive-provocative interactions predominantly induced anger (anger vs. serenity: *t* = 9.2, *p* < 0.00001; anger vs. happiness: *t* = 11.7, *p* < 0.00001; anger vs. fear: *t* = 5.6, *p* < 0.00001), followed by fear (fear vs. serenity: *t* = 2.0, *p* = 0.0574, as a statistical trend; fear vs. happiness: *t* = 3.8, *p* = 0.0007), and serenity (serenity vs. happiness: *t* = 4.0, *p* = 0.0004).

The neutral interactions were primarily categorized as leading to feelings of serenity (serenity vs. anger: *t* = 12.5, *p* < 0.00001; serenity vs. happiness: *t* = 4.7, *p* = 0.00007; serenity vs. fear: *t* = 15.0, *p* < 0.00001), followed by feelings of happiness (happiness vs. anger: *t* = 5.3, *p* = 0.00001; happiness vs. fear: *t* = 6.2, *p* < 0.00001), and anger (anger vs. fear: *t* = 1.9, *p* = 0.06, as a statistical trend). The social-positive interactions mainly resulted in happiness (happiness vs. anger: *t* = 28.9, *p* < 0.00001; happiness vs. serenity: *t* = 14.1, *p* < 0.00001; happiness vs. fear: *t* = 32.3, *p* < 0.00001), followed by serenity (serenity vs. anger: *t* = 6.1, *p* < 0.00001; serenity vs. fear: *t* = 7.7, *p* < 0.00001), and anger (anger vs. fear: *t* = 2.6, *p* = 0.01). Notably, although not supported by a significant interaction of GROUP × SC, the EG participants responded with happiness on neutral interactions more often than participants in the NG (M-U-Test: *Z* = 2.4, *p* = 0.02; see Figure 3B for details).

#### Decisions in the fMRI-Session

**Figure 4:**
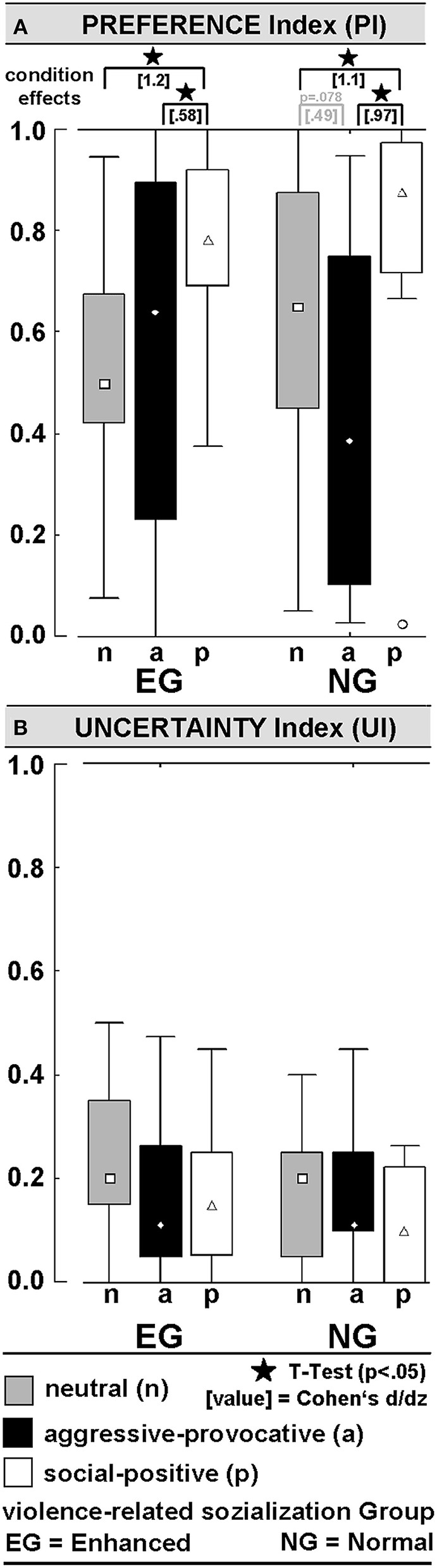
Behavioral data during functional MRI (fMRI): **(A)** Preference-Index (PI) indicates the level of pro-active engagement in the stimulus categories (= in no situation approach behavior was chosen; 1 = in all situations approach behavior chosen); **(B)** Uncertainty-Index (UI) indicates the percentage of inconsistent behavior in the repeatedly presented video clips (see methods section for more details).

PREFERENCE-INDEX (PI): The PI (0 ≤ PI ≤ 1) represents the ratio of trials in which the participant decided to respond with an approach behavior. An ANOVA (within the factors SC and PI, and between the factor GROUP) resulted in a significant main effect of SC [*F*_(2, 56)_ = 12.3, *p* = 0.00004]. Social-positive interactions produced higher PIs than aggressive-provocative (*t* = 4.2, *p* = 0.0002) and neutral (*t* = 6.3, *p* < 0.00001) interactions when the PIs were pooled for the EG and NG. Nevertheless, the group-specific profiles of PIs suggested that approach behaviors (i.e., reactive-aggressive responses) in the aggressive-provocative interactions were more often chosen in the EG than in the NG. However, the large distributions of PI-values (see Figure 4A) lowered the probability for the significant statistical discrimination of the EG and NG participants regarding reactive-aggressive responses to aggressive-provocative interactions. The group-specific data were separately illustrated in Figure 4A to provide a broader picture of the behavioral data, but of course, *post hoc* analyses should be cautiously interpreted and primarily used for generating hypotheses in future research. For instance, when exploring the individual differences in aggressive behaviors.

UNCERTAINTY-INDEX (UI): The UI (0 ≤ UI ≤ 1) represented the ratio of inconsistent (unreliable) decisions. Each video clip was presented twice during the fMRI session. Decisions were rated and counted as inconsistent if a participant decided differently after the repeated display of the very same video clip. Hence, the UI indicated how strongly a participant was inclined to respond in the same way to a specific interaction whenever this interaction was presented in a video clip. There were no interaction effects of the factors GROUP x SC or group differences on the UI. When pooling the data of both groups of participants, the UI-scores were statistically significantly different from a probability of 0.50: neutral vs. 0.5: *t* = 12.5, *p* < 0.00001; aggressive-provocative vs. 0.5: *t* = 13.8, *p* < 0.00001; social positive vs. 0.5: *t* = 15.7, *p* < 0.00001 (see Figure 4B for details). The latter results confirm that the decisions were not made by chance, but reliably related to the content of the video clip, and therefore, related to the social qualities of the displayed social interactions.

### fMRI Data

The fMRI activation clusters should be interpreted by keeping in mind that both the AROUSAL and KIND OF DECISION (i.e., approach and withdrawal) were modeled by additional regressors in the first level analyses (see also method section). Thus, these neural correlates indicate the brain regions that were involved in the decision-making in the complex social situations irrespective of the behavior chosen as a response (i.e., irrespective of the decision to approach or to withdraw from an interaction).

#### fMRI Contrasts for Decisions on Aggressive-Provocative Interactions (Contrast: Aggressive-Provocative vs. Neutral Interactions)

**Figure 5:**
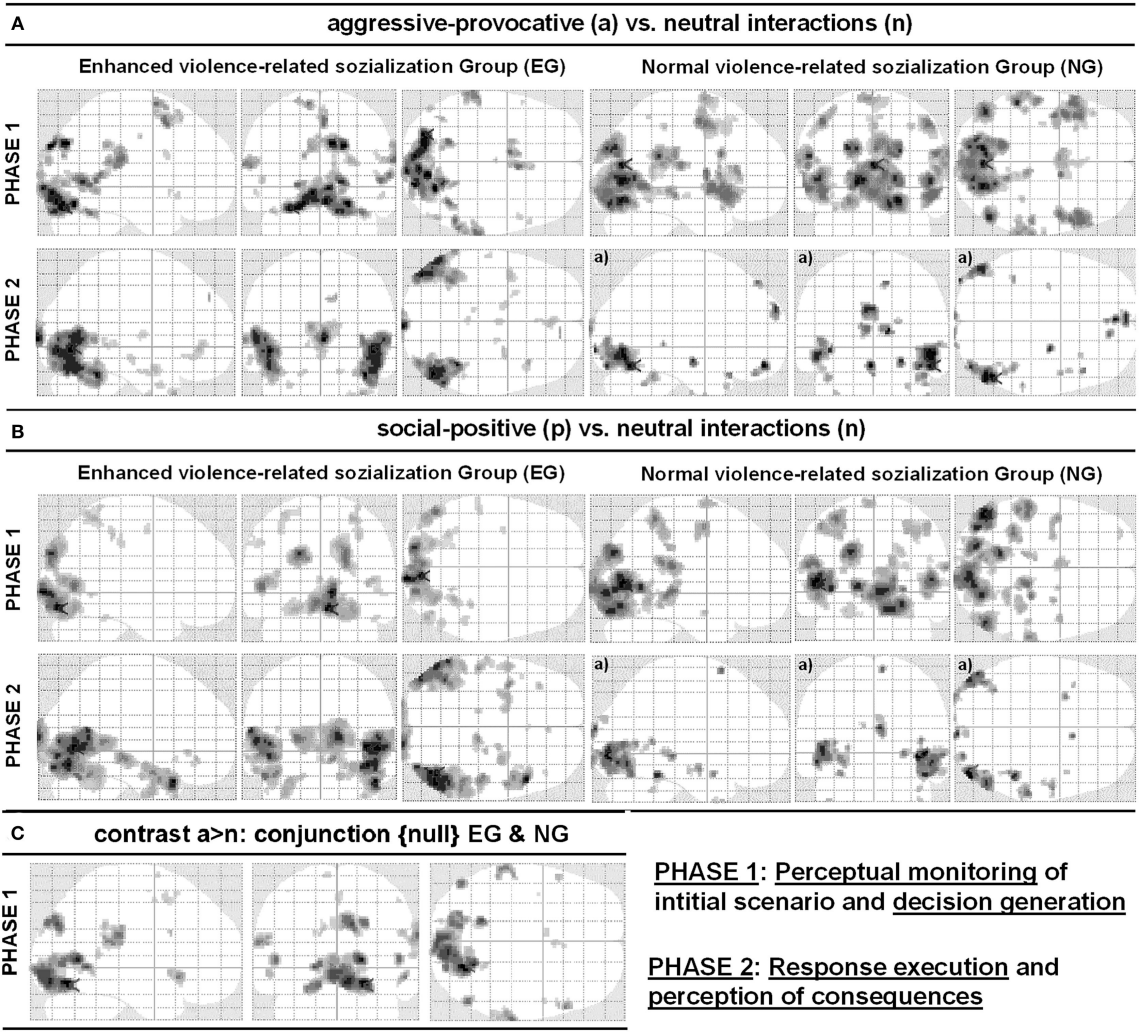
Glass-brain views of contrasts **(A)** social-conflict (reactive-aggressive) vs. neutral interaction and **(B)** social-positive vs. neutral interaction. **(C)** illustrates the respective conjunction {null} analyses including both groups (EG and NG), for the contrast social-aggressive-provocative (reactive-aggressive) versus neutral interaction for PHASE I video clips. The left part of the panels shows the results for the participant group with enhanced violence-related socialization (EG) and the right part of the panels shows results for the participant group with normal violence-related socialization (NG). Anatomical regions, peak activation t-values, and Talairach-coordinates are listed in Tables 2–4. All contrasts were *p* < 0.05, false discovery rate (FDR)-corrected, with a minimum cluster size of k = 5 voxels, except ^a)^: *p* < 0.001, uncorrected.

*PHASE I*: While watching the video clips and performing the corresponding appraisal of the displayed initial scenario (see Figure 1) as a content-related basis for the decision-generating process in aggressive-provocative interactions, the participants in EG produced activation patterns distributed as follows: in the superior and inferior frontal, cingulate, medial and lateral inferior parietal, left middle occipital and left and right medial occipital, bilateral fusiform, bilateral superior temporal and right middle temporal, and in bilateral cerebellar regions (see Table 2 for details and Figure 5A).

Table 1Post experimental (after MRI-scanning session) evaluation of the applied video stimuli according to arousal (11 level scale: 0–10), familiarity (11 level scale: 0–10), unambiguity (11 level scale: 0–10), the situation-related behavioral tendency (percentage of interactions, in which participants decided to withdraw from, to behave passively in or to approach the scenario), and situation-related emotional appraisal rating (percentage of interactions for which participants rated to felt angry, friendly, afraid or experience a neutral mood); standard deviations are presented in parenthesis (upper values: EG; lower values: NG).

**Table:** 

**Post experimental evaluation of the applied video stimuli**									
	**Arousal**			**Familiarity**			**Unambiguity**		
**Stimulus**	**Neutral**	**Social-positive**	**Aggressive-provocative**	**Neutral**	**Social-positive**	**Aggressive-provocative**	**Neutral**	**Social-positive**	**Aggressive-provocative**
	6.1 (1.6)	7.2 (1.9)	7.3 (2.2)	7.0 (1.3)	7.9 (1.2)	7.1 (2.1)	6.7 (1.7)	8.6 (1.1)	7.9 (1.8)
	4.7 (1.8)	6.3 (1.2)	7.7 (0.8)	6.8 (1.2)	8.0 (1.1)	4.6 (2.1)	6.5 (2.2)	8.7 (1.0)	8.2 (1.4)
**Behavioral tendency**									
	**Neutral scenario**			**Social-positive scenario**			**Aggressive-provocative scenario**		
	**Approach**	**Passive**	**Withdraw**	**Approach**	**Passive**	**Withdraw**	**Approach**	**Passive**	**Withdraw**
	37.0 (25.3)	36.3 (29.9)	26.7 (27.2)	64.0 (38.3)	21.0 (20.4)	15.0 (17.7)	59.0 (38.7)	19.0 (21.1)	22.0 (26.8)
	35.0 (27.2)	45.7 (22.0)	19.3 (18.5)	68.3 (24.1)	25.7 (22.8)	6.0 (7.6)	48.3 (33.9)	13.0 (7.7)	38.7 (31.5)

**Table:** 

**Emotional categorization (appraisal ratings)**											
**Neutral scenario**				**Social-positive scenario**				**Aggressive-provocative scenario**			
**Angry**	**Neutral**	**Friendly**	**Afraid**	**Angry**	**Neutral**	**Friendly**	**Afraid**	**Angry**	**Neutral**	**Friendly**	**Afraid**
8.7 (9.3)	52.0 (19.0)	36.0 (19.7)	3.3 (6.2)	3.0 (4.6)	14.0 (12.1)	82.3 (14.6)	0.7 (1.8)	66.7 (29.5)	12.0 (10.8)	5.0 (7.8)	16.3 (17.2)
8.7 (6.7)	63.0 (14.7)	21.3 (12.7)	7.0 (6.2)	2.0 (3.7)	19.7 (11.4)	77.7 (11.0)	0.3 (1.3)	62.3 (20.6)	11.3 (8.5)	3.7 (5.8)	22.7 (23.6)

**Table 2:** Anatomical regions, peak activation t-values, and Talairach-coordinates for contrasts (arranged in columns A–D) including the emotional stimulus category reactive-aggressive (a) and neutral (n) separately for the initial (a1 and n1) and the final part of the scenario (a2 and n2); H, hemisphere; L, left; R, right; B, bi-hemispheric; all statistics *p* < 0.05, FDR-corrected, minimum voxel cluster size k = 5 voxels; EG = participant group with enhanced violence-related socialization, NG = participant group with normal violence-related socialization; ^*^ cluster including Midbrain/PAG(peri-aqueductal gray); AMY = Amygdala.

		**Initial part of scenario (PHASE 1)**								**Final part of scenario (PHASE 2)**							
		**A**				**B**				**C**				**D**			
		**EG**				**NG**				**EG**				**NG**			
		**a1 > n1**				**a1 > n1**				**a2 > n2**				**a2 > n2**			
**Anatomical region**	**H**	**t**	**x**	**y**	**z**	**t**	**x**	**y**	**z**	**t**	**x**	**y**	**z**	**t**	**x**	**y**	**z**
**FMRI-contrasts for decisions in aggressive-provocative vs. neutral interactions**																	
Precentral gyrus	R					6.2	48	2	48								
Superior frontal gyrus	B					3.9	0	14	49								
	L	6.1	−10	7	64	5.0	−4	3	68					5.5	−2	62	26
	L	5.0	−18	7	66									4.1	−10	58	30
	R	6.6	4	12	49	3.9	12	9	64	5.2	14	50	36	4.9	12	47	47
	R	4.5	6	24	54												
	R	4.3	4	7	59												
Medial frontal gyrus	B									4.2	0	34	−10				
	L													5.8	−2	50	−16
	R					4.6	2	6	46					4.5	2	61	19
Middle frontal gyrus	L					6.1	−44	−1	50								
	L					4.7	−34	−1	59								
	L					3.9	−51	6	42								
Inferior frontal gyrus	L					7.0	−42	19	−4								
	R	4.3	51	13	23	7.0	55	19	−6								
	R	4.2	55	19	−8	6.1	55	15	25								
						5.2	55	28	12								
Orbital gyrus	B													4.7	0	42	−22
Paracentral lobule	R					3.7	8	−44	57								
Anterior cingulate	B									4.7	0	41	−4				
	L									4.1	−2	27	−8				
Cingulate gyrus (central part)	L	4.3	−2	−8	39												
	R					3.9	2	21	41								
Posterior cingulate	R	7.5	20	−60	7												
	R	4.6	8	−64	11												
Insula	L					6.8	−44	6	−2								
Postcentral gyrus	L					5.9	−57	−19	18								
	R					4.3	34	−31	48								
	R					4.0	65	−13	17								
Inferior parietal lobule	L					7.1	−55	−38	26								
	R	6.2	67	−24	23	5.5	67	−24	25								
	R	5.2	65	−37	33	5.3	65	−37	28								
	R	4.4	55	−30	22												
Precuneus	R	8.3	12	−72	37	9.5	2	−69	20								
Middle occipital gyrus	L	4.4	−50	−69	9	7.5	−48	−69	9	9.0	−44	−80	1	5.5	−48	−72	−3
	L									8.1	−44	−72	−8	5.5	−38	−93	1
	L													4.8	−46	−83	8
	R									8.8	50	−70	−8	6.0	51	−63	−10
	R													5.9	50	−68	−5
	R													5.9	14	−99	18
	R													4.4	22	−96	23
Inferior occipital gyrus	R													5.3	42	−84	−9
Cuneus	L	5.0	−14	−75	6	9.0	−12	−69	9	5.6	−8	−99	20	5.0	−4	−98	20
	R									9.0	4	−97	10				
	R									4.8	10	−98	20				
Lingual gyrus	L	6.4	−18	−54	5												
	L	4.7	−14	−58	0												
	R	5.1	20	−49	−1	8.4	24	−72	−6								
Fusiform gyrus	L	8.4	−26	−76	−11												
	R	8.2	24	−66	−8					8.9	40	−48	−18				
Superior temporal gyrus	L	5.8	−63	−30	18	6.7	−53	12	−2	4.9	−42	3	−17				
	L	4.2	−55	13	−6	5.0	−63	−34	20								
	R	5.9	61	−44	11	6.4	51	10	−4	5.4	36	18	−35	4.8	44	20	−21
	R					5.1	65	−36	18	4.7	40	−39	4	4.1	30	16	−29
	R									4.2	50	−40	8				
Middle temporal gyrus	L									8.6	−55	−64	9				
	L									4.4	−59	−16	−8				
	R	6.1	53	−48	6	8.0	51	−66	9					4.6	57	−35	4
	R	4.7	51	−64	11	5.4	55	−26	−5					4.1	46	6	−42
	R	4.1	50	−71	15												
Inferior temporal gyrus	R									9.9	46	−68	2	4.5	48	−2	−37
Parahippocampal gyrus	L									4.2	−16	−3	−18				
	R									4.3	16	−11	−18				
Parahippocampal gyrus/AMY	R													5.4	24	−10	−10
Uncus	L									4.2	−28	6	−27	4.3	−32	−13	−33
	R									4.5	26	−6	−35				
Thalamus	B									4.4	0	−7	10				
	L									4.4	−12	−31	3				
Thalamus, adjacent to Caudate Body, Putamen, Globus Pallidus	L					6.9	12	2	9								
Claustrum	L					4.0	−36	−16	−6								
Brainstem/Midbrain*	L									4.2	−2	−37	2				
Cerebellum/Culmen	L													4.5	−40	−44	−20
	R													4.5	44	−46	−20
Cerebellum/Tuber	R													4.5	46	−54	−24
Cerebellum/Declive	L	4.5	−40	−61	−15												
	R	5.9	36	−55	−16												

The participants in the NG produced the following activation patterns under the same conditions: in the precentral, bilateral superior, right medial, left middle, and bilateral inferior frontal, right paracentral, bilateral postcentral, right inferior and medial parietal, left middle and bilateral medial occipital, bilateral superior, and right middle temporal regions. Additionally, the brain activation patterns observed in the participants of the NG involved a thalamic region adjacent to the left caudatum, putamen, and globus pallidus, and the left claustrum (see Table 2 for details and Figure 5A).

*PHASE II*: For the execution phase of decision-making (i.e., pressing a response key and watching/assessing the consequences of the decisions, see Figure 1) in aggressive-provocative interactions, the EG showed activation patterns in the following brain areas: the right and bilateral medial frontal, bilateral anterior cingulate, bilateral middle and medial occipital, right fusiform, bilateral superior, left middle and right inferior temporal brain regions, and bilateral in the parahippocampal gyri. Further, the brain activation patterns in the bilateral uncus and thalamus, and the left brainstem were observed (see Table 2 for details and Figure 5A).

The participants in the NG produced activation patterns in the bilateral superior, medial and orbital frontal, in the bilateral middle, right inferior, and left medial occipital, and in the right superior, middle, and inferior temporal brain regions. Additionally, they recruited the right parahippocampal gyrus, adjacent to the amygdala, the left uncus, and bilateral cerebellar areas (see Table 2 for details and Figure 5A).

#### fMRI Contrasts for Decisions on Social-Positive Contexts (Contrast: Social-Positive vs. Neutral Interactions)

*PHASE I*: While watching the video clips and performing the corresponding appraisal of the displayed initial scenario (see Figure 1) as a content-related basis for the decision-generating process in the social-positive scenarios, the EG produced activation patterns distributed in the following brain regions: the left middle frontal, bilateral post-central, bilateral superior and left inferior parietal, right middle and bilateral medial occipital, and bilateral middle and in right inferior temporal regions (see Table 3 for details and Figure 5B).

**Table 3:** Anatomical regions, peak activation t-values, and Talairach-coordinates for contrasts (arranged in columns A–D) including the emotional stimulus category social positive (p) and neutral (n) separately for the initial (p1 and n1) and the final part of the scenario (p2 and n2); H, hemisphere; L, left; R, right; B, bi-hemispheric; all statistics *p* < 0.05, FDR-corrected, minimum voxel cluster size k = 5 voxels; ^a)^ *p* < 0.001, uncorrected; EG = participant group with enhanced violence-related socialization, NG = participant group with normal violence-related socialization; ^*^ cluster including midbrain/PAG (Peri-Aqueductal Gray).

		**Initial part of scenario (PHASE 1)**								**Final part of scenario (PHASE 2)**							
		**A**				**B**				**C**				**D**			
		**EG**				**NG**				**EG**				**NG**			
		**p1 > n1**				**p1 > n1**				**p2 > n2**				**p2 > n2 ^a)^**			
**Anatomical region**	**H**	**t**	**x**	**y**	**z**	**t**	**x**	**y**	**z**	**t**	**x**	**y**	**z**	**t**	**x**	**y**	**z**
**FMRI-contrasts for decisions in social-positive vs. neutral interactions**																	
Superior frontal gyrus	L					4.8	−4	1	68								
Middle frontal gyrus	L	4.4	−36	−1	48	5.6	−46	1	50								
	L					5.0	−30	−4	46								
	R					5.4	30	−3	48								
	R					5.0	48	3	51								
Inferior frontal gyrus	L									4.6	−38	29	−1				
	R									3.8	42	34	−10				
Orbital gyrus	L					4.0	−32	54	−43								
Anterior cingulate	R									4.8	2	36	−9				
	R									3.8	2	17	−8				
Cingulate gyrus (central part)	L					5.2	−12	−29	35								
Posterior cingulate (PC)	L					8.1	−14	−56	8								
Insula	R					4.3	50	−28	18								
Postcentral gyrus	L	4.5	−51	−23	38	6.3	−32	−40	59								
	L	4.2	−18	−47	65	4.8	−26	−34	51								
	L	4.0	−57	−25	44												
	R	4.9	34	−33	46	6.1	63	−28	20								
	R	4.6	34	−41	68	4.9	36	−33	48								
	R					4.7	30	−34	53								
Superior parietal lobule	L	5.3	−30	−53	63												
	R	5.6	30	−48	59												
Inferior parietal lobule	L	4.3	−32	−46	54	7.0	−50	−28	24								
	L	4.3	−63	−27	38	6.3	−57	−28	22								
	L					5.9	−53	−38	26								
	R					4.0	50	−28	27								
Precuneus	L					7.4	−22	−74	35								
	R					4.4	2	−61	18								
	R					4.2	20	−72	39								
Middle occipital gyrus	L					9.5	−50	−69	9	5.5	−12	−99	16	6.1	−46	−38	8
	L					8.0	−38	−77	6	7.9	−48	−79	11	5.7	−50	−76	−3
	L					4.3	−26	−88	21								
	R	4.2	30	−81	17	5.5	46	−74	2	10.0	42	−66	5	6.7	40	−83	2
	R					4.5	46	−79	9	9.1	50	−68	5	5.8	50	−68	−5
	R									8.8	16	−98	16	5.6	26	−89	4
Inferior occipital gyrus	R									9.0	42	−72	−6				
Cuneus	B	4.8	0	−81	13									3.8	0	−88	23
	L	10.1	−18	−84	34					6.7	−20	−99	14	4.3	−8	−98	25
	R	12.9	10	−95	5	4.3	24	−82	35					5.8	6	−94	23
	R	8.6	22	−84	32									4.4	6	−84	30
Lingual gyrus	L					4.2	−24	−72	−10								
	L					4.0	−22	−82	−6								
	R	13.2	8	−80	−8	8.2	8	−83	4								
	R	8.9	12	−86	−2	8.1	14	−80	−8								
	R	5.0	20	−52	1												
Fusiform gyrus	L					4.1	−22	−80	−13	7.3	−48	−63	−15				
	L									6.3	−40	−55	−16				
	L									5.0	−44	−47	−11				
	R													5.3	50	−65	−12
	R													6.2	46	−43	−13
Superior temporal gyrus	L									6.3	−38	−1	−15	4.3	−38	10	−34
	L									3.7	−46	12	−29				
	R									9.2	48	16	−23	5.0	42	−46	12
	R									6.1	46	5	−20				
	R									6.1	46	−24	−6				
Middle temporal gyrus	L	8.8	−51	−66	9	6.9	−42	−66	11	8.5	−55	−66	11	5.4	−42	−71	11
	L	6.5	−46	−75	13					8.3	−44	−58	8				
	L	5.1	−36	−81	19					5.6	−53	−27	−2				
	L									4.6	−63	−20	−4				
	L									4.0	−57	−16	−8				
	L									5.3	−55	1	−14				
	L									4.0	−55	−8	−13				
	L																
	R	5.8	46	−73	13	7.3	50	−69	13	5.6	−38	−65	20	5.2	61	−29	0
	R	4.7	50	−60	5	3.8	65	−48	8					4.8	55	−6	−11
	R													4.5	59	−37	2
	R													4.2	42	6	−36
	R													4.1	50	−16	−13
	R													3.9	42	−56	5
	R													3.9	63	−8	−11
Inferior temporal gyrus	R	4.8	48	−40	−17												
	R	4.3	55	−64	2												
Uncus	L									5.3	−30	−1	−29				
	L									5.2	−26	−1	−22				
Parahippocampal gyrus (PHG)*	L									4.8	−16	−3	−18	4.8	−28	5	−15
	R					5.0	18	−39	−6	4.9	18	−11	−18				
	R					4.9	26	−49	−3	4.5	22	−22	−9				
	R					4.7	18	−3	−13								
Between PC and PHG	R					4.2	20	−48	8								
Between PHG and Amygdala	R									5.1	28	−6	−11				
Thalamus	L					4.5	−8	−23	9	4.3	−14	−27	0				
	R					6.8	14	−29	1	4.0	20	−29	1				
	R					6.3	26	−27	3								
Brainstem/Midbrain*	R									5.8	10	−27	−5				
Cerebellum/Culmen	R													4.3	40	−48	−25

For the same contrast between conditions, the participants in the NG produced activation patterns in the left superior, bilateral middle, and left orbital frontal, in the left central and posterior cingulate, right insular, bilateral postcentral, bilateral inferior and medial parietal, in the bilateral middle and medial occipital, in the left fusiform, and bilateral middle temporal brain regions. Additionally, the brain activation patterns in the NG involved a cluster of regions including the right parahippocampal areas, the PAG, and the bilateral thalamus (see Table 3 for details and Figure 5B).

*PHASE II*: For the execution phase of decision-making (i.e., pressing a response key and watching/assessing the consequences of the decisions, see Figure 1) in social positive interactions, the EG showed activation patterns in the following brain areas: the bilateral inferior frontal, right anterior cingulate, bilateral middle, right inferior, and left medial occipital, left fusiform, and in bilateral superior and middle temporal brain regions. Additionally, for this group of participants, we found brain activations in the bilateral parahippocampal gyri, in the bilateral thalamus, and the brainstem, adjacent to the PAG. Further, the NG produced activation patterns in the bilateral middle and medial occipital, in the right fusiform, in the bilateral superior and middle temporal, in the parahippocampal, and right cerebellar brain regions (see Table 3 for details and Figure 5B).

#### Conjunction Analysis Including fMRI Contrasts for Decisions on Aggressive-Provocative Interactions (Conjunction {Null} EG and NG)

When contrasting aggressive-provocative vs. neutral interactions in a conjunction analysis (conjunction {null}; cf., Friston et al., 2005) that included the EG and the NG, the brain activation patterns distributed in the frontal, parietal, occipital, and temporal iso-cortex were observed. Particularly involved were the bilateral superior and middle, right medial and inferior frontal gyri, the right posterior cingulate cortex, left postcentral gyrus, right inferior parietal lobule and bilateral precuneus, left middle occipital gyrus, bilateral medial occipital areas in the cuneus and lingual gyri, right fusiform gyrus, and the bilateral superior and right temporal gyri (see Table 4 for details and Figure 5C).

**Table 4:** Anatomical regions, peak activation t-values, and Talairach-coordinates for conjunction {null} analysis “EG and NG” including contrast images for aggressive-provocative (a1) vs. neutral (n1) stimulus condition for the initial PHASE I; H, hemisphere; L, left; R, right; statistics: *p* < 0.05, FDR-corrected, minimum voxel cluster size k = 5 voxels; EG = participant group with enhanced violence-related socialization, NG = participant group with normal violence-related socialization.

**Anatomical region**	**H**	**t**	**x**	**y**	**z**
**FMRI conjunction analysis (PHASE I)**					
**Aggressive-provocative vs. neutral interaction condition (EG and NG)**					
Superior frontal gyrus	L	3.8	−10	7	66
	R	3.7	4	20	52
Medial frontal gyrus	R	4.0	2	12	47
Middle frontal gyrus	L	3.6	−40	−1	55
	R	3.7	51	4	48
Inferior frontal gyrus	R	4.1	53	15	25
	R	4.7	55	19	−8
Posterior cingulate	R	6.0	20	−60	9
	R	4.3	8	−64	11
Postcentral gyrus	L	4.5	−59	−26	20
Inferior parietal lobule	R	5.0	67	−26	25
	R	4.2	65	−37	33
Precuneus	L	3.9	−2	−72	35
	R	5.6	12	−74	35
Middle occipital gyrus	L	5.3	−50	−69	9
Cuneus	L	4.0	−12	−74	30
	L	3.7	−18	−86	30
	R	5.4	24	−82	35
	R	3.6	18	−88	30
Lingual gyrus	L	5.8	−2	−89	−1
	L	5.5	−18	−52	3
	R	6.2	8	−82	−1
	R	4.2	18	−49	−1
Fusiform gyrus	R	6.7	22	−66	−8
Superior temporal gyrus	L	4.8	−61	−36	20
	L	4.3	−55	13	−4
	R	4.0	63	−44	11
Middle temporal gyrus	R	5.3	51	−64	9

#### Regions of Interest Analyses for PHASE I

**Figure 6:**
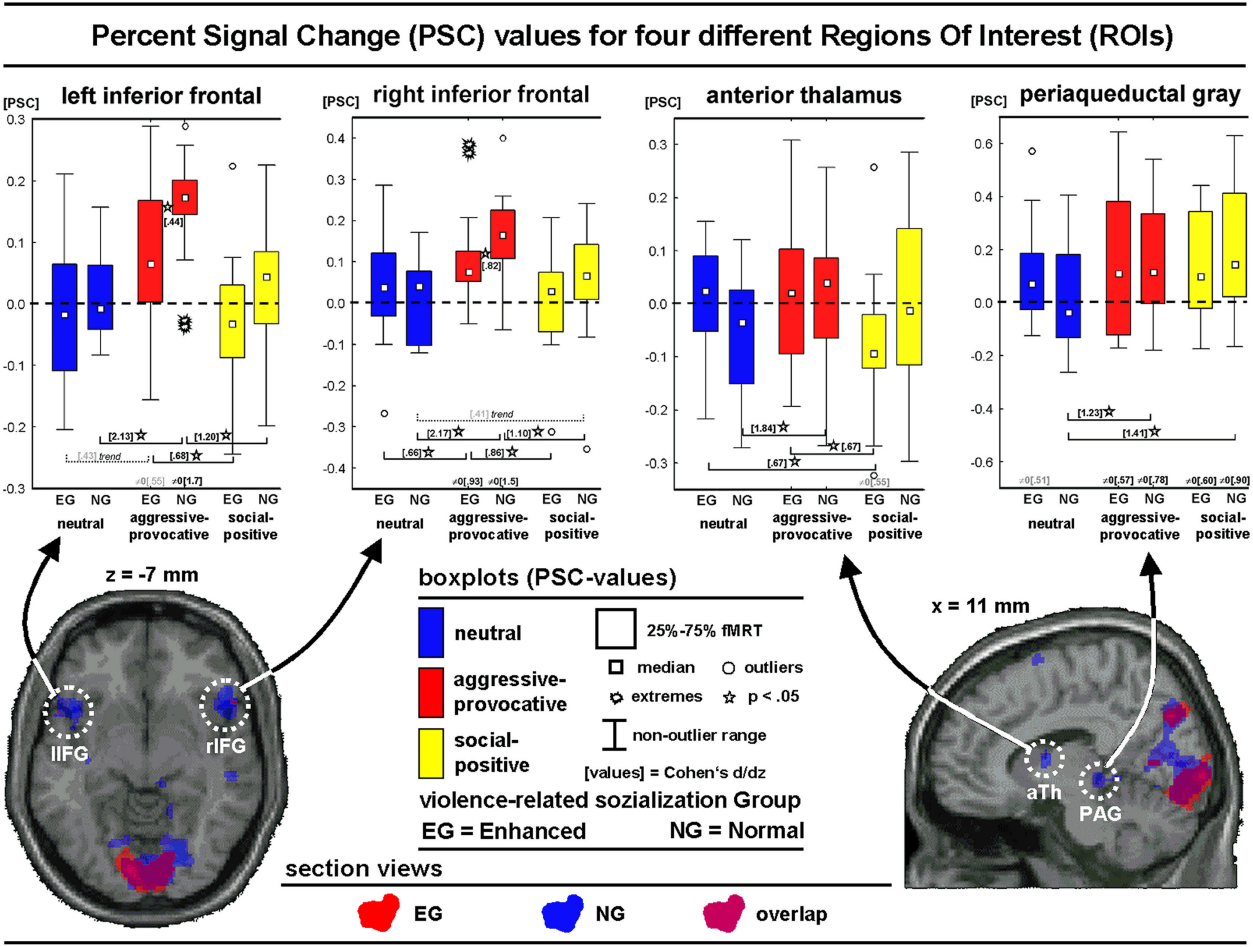
Regions of interest analyses showing section views and box-plots of four regions of interest (ROIs) illustrating the distribution of the percent signal change (PSC) values (see text for more details) of the different emotional categories for the initial phase (PHASE I) of the interactions: Neutral, social-aggressive-provocative, and social-positive.

The percent signal change (PSC) values were extracted from four ROIs oriented on predefined regional hypotheses (i.e., left and right inferior frontal, thalamus, and PAG) and cluster-wise specifications based on a second-level group analysis in the NG (contrast: PHASE I, decisions on aggressive-provocative vs. neutral interactions). The PSC values should be handled with care as the convolution of slow hemodynamic responses might have contaminated the discriminatory value of this parameter. In the present study, we modeled short trial elements, but we also applied a non-stationary probabilistic trial sequence (Friston, 2000), supposed to enhance the validity of the extracted PSC values. The boxplots, statistics, and section views of the ROIs are illustrated in Figure 6.

For the *left inferior frontal* ROI, the main effect of the SC was found [*F*_(2, 56)_ = 17.1, *p* < 0.00001; GG: *p* < 0.00001]. A *post hoc* Kolmogorov-Smirnov-Test (K-S-T) indicated larger PSC values for the NG compared with the EG for the aggressive-provocative SC (K-S-T: *p* < 0.05). The group-unspecific SC-effects (Wilcoxon-Matched-Pairs-Test; W-M-P-T) revealed higher PSC values for the decisions in the aggressive-provocative compared with social-positive as well as with neutral interactions in both the EG (Z = 2.7, *p* < 0.01 and Z = 1.8, *p* = 0.08, as a trend to significance) and the NG (Z = 3.2, *p* < 0.01 and Z = 3.4, *p* = 0.0007). The PSC values for the decisions in the aggressive-provocative interactions exceeded zero for both the EG and NG (EG: *t* = 2.1, *p* = 0.05; NG: *t* = 6.6, *p* = 0.00001).

For the *right inferior frontal* ROI, a trend of significance for the interaction-effect for GROUP × SC [*F*_(2, 56)_ = 2.6, *p* = 0.08; GG: *p* = 0.09] and a significant main effect for SC [*F*_(2, 56)_ = 23.1, *p* < 0.00001; GG: *p* < 0.00001] were revealed. A *post hoc* test indicated higher PSC values for the NG compared with the EG for the aggressive-provocative SC (K-S-T: *p* < 0.05). The group-unspecific SC effects revealed larger PSC values for the aggressive-provocative compared with social-positive and neutral interactions in both the EG (W-M-P-T: Z = 3.1, *p* = 0.002; Z = 2.0, *p* = 0.04) and the NG (W-M-P-T: Z = 3.0, *p* = 0.003; Z = 3.4, *p* = 0.0007). The PSC values for the aggressive-provocative interactions exceeded zero in both the EG and the NG (EG: *t* = 3.6, *p* < 0.003; NG: *t* = 5.7, *p* < 0.00006).

An analysis of the *anterior thalamic* ROI showed the significant interaction of GROUP x SC [*F*_(2, 56)_ = 4.9, *p* = 0.01; GG: *p* = 0.01) and the main effect for SC [*F*_(2, 56)_ = 4.6, *p* = 0.01; GG: *p* = 0.02). The group-specific SC effects revealed higher PSC values for the aggressive-provocative and neutral compared to the social-positive interactions in the EG (W-M-P-T: Z = 2.1, *p* = 0.04; Z = 2.2, *p* = 0.03) and aggressive-provocative compared with the neutral interactions in the NG (W-M-P-T: Z = 3.4, *p* = 0.0007). A statistical trend indicated PSC values less than zero for the social-positive interactions in the EG (*t* = 2.1, *p* = 0.05).

An analysis of the ROI PAG showed the significant interaction of GROUP × SC [*F*_(2, 56)_ = 6.6, *p* = 0.002; GG: *p* = 0.003] and the main effect of SC [*F*_(2, 56)_ = 12.5, *p* = 0.00003; GG: *p* = 0.00004]. The group-specific SC-effects revealed higher PSC values for the aggressive-provocative and social-positive compared with the neutral interactions in the NG (W-M-P-T: Z = 3.0, *p* = 0.003, Z = 3.3, *p* = 0.001). The PSC values for the aggressive-provocative and social-positive interactions exceeded zero in both the EG and the NG (EG: *t* = 2.2, *p* = 0.05, and; *t* = 2.3, *p* = 0.04; NG: *t* = 3.0, *p* = 0.009, and *t* = 3.5, *p* = 0.004). Moreover, the PSC values statistically tended to exceed zero for the neutral interactions in the EG only (*t* = 2.0, *p* = 0.07).

## Discussion

The neural correlates of decision-making in complex social situations were investigated in the MR scanner tube by presenting video clips that showed realistic interactions between the observer (participant) and other persons. The participants were individuals with normal socialization on violence (NG), and persons with socialization histories characterized by heightened levels of violent experiences (EG). Thirteen EG participants were members of the open regime, and two of them were members of the closed regime at the local correctional and rehabilitation center. Our video clips showed aggressive-provocative, social-positive, and neutral interactions from a first-person perspective. These interactions were supposed to induce negative emotions (anger or fear), positive emotions (friendliness), or an affectively neutral mood. The expected emotional responses to these social interactions were validated by the categorization of the participants of the emotions they experienced while watching the video clips after the fMRI experiment. During the MRI scanning session, the participants were asked to get deeply involved in the displayed interactions. The participants also were requested to participate actively in the interactions by choosing between the approach and withdrawal behavior as a response to the interaction they watched.

The neural correlates of the decisions of the participants on these realistic social interactions were explored separately for the EG and the NG. The regions of interest analyses confirmed our hypotheses that the midbrain structures (i.e., the PAG) were recruited for the processing of aggressive provocative interaction scenarios. Additionally, the EG showed both less distributed and less intense inferior frontal brain activation (i.e., in the left and right inferior frontal gyri) while processing the aggressive-provocative interactions compared with the NG. Note that the NG consisted of individuals with normal socialization, which means that they did not experience much violence while growing up. In addition, the participants in the NG did not have a history of delinquency.

### The Influence of Different Socialization Backgrounds and Their Consequences for Social Decision-Making in Quasi-Realistic Interaction Scenarios

Statistically, both groups of participants (i.e., the EG and NG), made similar decisions on the three kinds of social interactions in the fMRI experiment. In other words, they showed comparable behavioral responses to these interactions. However, in contrast with the participants in the EG, the NG participants had a bi-modal profile in their decisions. The NG significantly chose to approach social interactions and neutral interactions more often (showing a considerable mean effect size; cf. Cohen, 1988) as compared with the aggressive-provocative interactions than the EG. Further, participants in the EG more frequently decided to approach an interaction both in the aggressive-provocative and social-positive interactions compared with neutral interactions.

Note, that in both groups, the unambiguity ratings of the neutral interactions were lower compared with the aggressive-provocative and social-positive interactions. These ratings indicated that the interpretation of the aggressive-provocative and social-positive interactions were rather clear in both groups of participants, while the interpretation of neutral interactions was less clear. This observation, together with the finding for the EG that the scores of the UI (consistency of decisions on an interaction displayed in a video clip) were skewed to the right for the decisions on neutral interactions, suggested that the EG made more inconsistent decisions regarding the neutral social interactions than the NG. We interpret the inconsistent decisions in the neutral interactions in the EG as an indicator that responding to neutral social situations is rather uncertain. This might be due to inefficient self-regulation strategies and/or low levels of self-control in this group of participants. In line with this argument, previous research claimed that two flawed self-regulation strategies (i.e., to over-and to under-regulated emotional responses) might be a very important cause for destructive aggressive behavior (see Roberton et al., 2012). The problems with self-regulation often come along with unstable social relationships and/or exaggerated uncertainty in a neutral social context, that might potentially be interpreted as threatening. The latter idea was also underpinned by a tendency to a generally elevated PAG involvement in the neutral interaction scenarios in the EG. Further, we speculate that the lack of active social problem-solving skills might have strongly supported the elevated frequency of aggressive behaviors in individuals with an above-average level of experiences of violence during their socialization (see also Lawrence and Hodgkins, 2009).

In the post-experimental evaluation ratings of the video clips, both groups of participants mostly chose the approach behavior as a response to the social-positive interactions. These decisions supported the validity of our video clip inventory (cf., Fehr et al., 2014; Fehr and Achtziger, 2021). As expected, in the aggressive-provocative interactions, the EG tended to respond with approach (i.e., they indicated reactive aggression). In contrast, the NG showed an ambivalent bi-modal preference for approach and withdrawal behavior. This might be expected in terms of a normal response toward different scenarios of threat (cf., Ramirez and Andreu, 2006).

A careful analysis of the post-experimental affective ratings of the interactions revealed some overlap between the results of both groups of participants. The scenarios provoking reactive-aggressive behavior were rated as inducing either anger or fear, with a clear preference for anger in all the participants (Ramirez and Andreu, 2006). This finding highlighted the validity of the aggressive-provocative interactions displayed in a subgroup of our video clips as a method useful for anger induction (Fehr et al., 2007, 2014; Strüber and Fehr, 2009; Fehr, 2012; Fehr and Achtziger, 2021). Interestingly, the bi-modal distribution of anger and fear categorizations did correspond to the bi-modal distribution of the approach and withdrawal decisions only in the NG, but not in the EG (see above). The dominance of approach decisions as responses toward the aggressive-provocative scenarios irrespective of the fear or anger categorizations of emotions in the EG supported the idea of a dysfunctional behavioral regulation in this group of individuals. In other words, socialization shaped by an above-average experience of violence might help to associate the emotion of fear with approach behavior (e.g., reactive aggression or fight) rather than with withdrawal behavior (i.e., flight).

The EG showed higher arousal ratings toward neutral interactions compared with the NG, which might be due to feelings of general distrust in social contexts (Lawrence and Hodgkins, 2009). This finding also fitted well to the socialization of the participants in the EG.

Also important for the interpretation of our findings was that the two groups of participants did not differ in the familiarity ratings of the neutral and social-positive scenarios. But note that the EG reported being more familiar with the aggressive-provocative interactions than the NG. This finding supported our claim that the aggressive-provocative interaction scenarios were of high levels of ecological validity as they clearly distinguished both groups of participants. These findings should be kept in mind when interpreting our physiological data as they highlight our argument that we measured the neural correlates of decision-making on how to act in highly realistic social interactions. Due to the purpose of the present research to investigate the neural correlates of aggressive behavior, physiological responses to conflict-laden interactions, like the ones displayed in aggressive provocative video clips, were of the highest interest, especially among our participants, who experienced violence relatively frequently during their socialization (i.e., the EG).

### Neural Correlates of Decisions on Aggressive-Provocative Interactions Depending on Participants' Socialization to Violence

When interpreting the group-related fMRI data concerning the decision toward the interactions presented in the video clips (i.e., the behavioral data), it should be kept in mind that in the fMRI data analyses at the first level (individual modeling), the arousal ratings (on a ten-point Likert-scale) and the type of decision (0 = withdrawal, 1 = approach) were added as regressors to the design matrices. Thus, the fMRI activation patterns were controlled for the arousal and the type of decision made in the scanner session. The variable of interest in the fMRI data was the neural correlates of the decisions and the question if these correlates differed between the two groups of participants, as they were socialized differently with regards to violence. For the analyses of the fMRI data, it did not matter what kind of decision a person made, but rather what group of participants an individual belonged to.

Conjunction analyses suggested that in conflict (i.e., aggressive-provocative) situations, in general, all the brain regions that were recruited in the NG were also recruited in the EG. From the perspective of brain physiology, it appeared that the brains of both groups of participants were involved in the same decision processes in the interactions that can lead to reactive-aggressive behavior. In other words, the brains of the individuals in both groups appeared to principally recruit the same structures to make decisions on the different emotional types of interaction categories. However, in the aggressive-provocative interactions in contrast with neutral interactions, the NG produced *more extended* activation clusters in the left and right inferior frontal brain regions than the EG. This is highly understandable as these regions were frequently discussed as the promising candidates for the top-down control of highly emotional and in particular of impulsive types of behavior (Ochsner and Gross, 2005; Strüber et al., 2008).

This argument was further supported by the ROI analyses revealing the signal intensity in the inferior frontal regions. The PSC values should be interpreted cautiously in the fMRI studies, yet, our experimental design with its non-stationary probabilistic weighted sequencing of the stimulus material (i.e., the video clips) should have increased the validity of this parameter significantly. Indeed, the ROI analyses yielded larger PSC values for the decisions toward the aggressive-provocative compared with social-positive and neutral interactions in both groups of participants. Note that this effect was stronger in the NG. This finding indicated the recruitment of more neural resources (quantitatively as suggested by the PSC value estimates, and qualitatively as implied by the extension of activation clusters) involved in action control in conflict situations among the participants with normal socialization to violence. If this interpretation is correct, it suggests that individuals with histories of delinquency might have problems in activating inferior frontal resources that are necessary for the appropriate control of violent impulses (cf., Brower and Price, 2001; Dolan, 2010). This should especially be the case if both a certain threshold of provocation is exceeded, and situational factors facilitating violence are present. Violent behavior might become more probable under these conditions in individuals with reduced neural recruitment of the inferior frontal brain areas responsible for the top-down control of behavior.

These results further indicated that the brain regions potentially responsible for the bottom-up processes (e.g., the anterior thalamus and the brainstem) appeared to be differentially involved in affectively modulated decision processes underlying complex social interactions. The EG produced relatively smaller PSC values in the anterior thalamus when making decisions toward social-positive compared with aggressive-provocative and neutral interaction scenarios while controlling for arousal by adding it as a regressor to the fMRI first-level analyses. The NG, on the other hand, produced relatively smaller PSC values in the anterior thalamus when making decisions on the neutral compared with aggressive provocative interaction scenarios.

Both groups (EG and NG) produced similar PSC values when deciding on how to respond to the provocative aggressive or positive social interaction scenarios (i.e., how to respond to affective social situations). These values differed from zero. However, only the EG produced larger PSC values when making decisions regarding the neutral interaction scenarios as indicated by a statistical trend with a medium effect size (cf., Cohen, 1988) that was comparable to the effect sizes of the other interaction categories. Furthermore, only the NG did not show statistically different PSC values between the interaction categories. Whereas, this effect has to be interpreted with caution and with respect to the complex nature of the examined parameters in the present study, this finding supported the idea that the processes of sub-cortical bottom-up regulation of decisions in social situations are generally more pronounced, at least, in a considerable number of individuals with a violent socialization history. Further, this observation might reflect hyper-sensitization and/or general distrust in social interactions in people with relatively frequent experiences of violence in their socialization (see Lawrence and Hodgkins, 2009). The latter conclusion should however be further investigated and differentiated by subsequent studies.

Summarizing, we showed that the control of the impulsive types of aggressive behavior might be substantially modulated by the inferior frontal iso-cortical (top-down control) and sub-cortical brain regions (i.e., by the bottom-up control of behavior in affective contexts through their evaluation or reflexive impulsive behaviors; see Nelson and Trainor, 2007; Strüber et al., 2008; Fehr, 2012; Fehr et al., 2014). In addition, our research suggested the frontal top-down-regulation of emotions by a brain mechanism potentially responsible for the self-control of behaviors in individuals with frequent experiences of violence in their socialization and with a history of delinquency (Brower and Price, 2001; Strüber et al., 2008; Wahl, 2009; Dolan, 2010; Roberton et al., 2012 for overviews).

As the participants in the EG did not perform open violent behaviors in the scanner, they controlled their aggressive impulses. This suggested that the participants in the EG can control their aggressive impulses, at least in an experimental setting. One could speculate that the ability to temporarily overregulate their affective impulses in cases where the situation requires it, might be a source of negative inner states (e.g., anger, sadness). These negative states can then lead to open aggression in situations that do not impose strong pressure on emotional control (see Roberton et al., 2012; and see discussion above). The peculiarities in brain activation patterns observed in the EG, therefore, shed light on the neural organization of the overregulation mechanisms of emotions that can temporarily be activated in individuals with socialization shaped by frequent experiences of violence. These peculiarities might, at least in part, be summarized as follows:

1. A lesser extent of the inferior frontal activations in the EG might point to lower levels of self-regulation reflected at the neural level (cf., Knoch and Fehr, 2007). But, as the EG did not show open violent behaviors in the scanner, it appeared that they were able to recruit other neural circuits to control their impulses at least for a while (see below). Alternatively, their aggressive impulses become much stronger in real-life situations than in experimental settings, and hence, the participants of the EG are not able to control themselves as they are overwhelmed. In addition, being watched by scientists in the fMRI laboratory increases self-awareness, which leads to behaviors that are in accordance with social norms.
2. The missing activation patterns in the middle frontal regions in the EG compared with the NG could reflect the lower executive control skills in this group of participants. The lack of the functions of these skills might be partially compensated by recruiting the superior frontal brain regions. This argument was strengthened by the activation patterns observed in these brain areas when the participants got involved in the aggressive-provocative interactions. In previous research, the dorsal sections of the frontal brain such as the superior frontal brain regions were associated with high cognitive control, whereas the ventral frontal brain regions such as the inferior frontal gyri were directly associated with emotional regulation (e.g., Davidson et al., 2000; Ochsner and Gross, 2005). Thus, individuals with reduced emotional control skills might be prone to use higher cognitive top-down control mechanisms to successfully regulate their impulsive behavior tendencies temporarily, but not across situations and not for a longer period. Additionally, a more idiosyncratic organization of the inferior frontal brain recruitment might also lead to dysfunctional consequences in behavioral control in the EG, which might be underpinned by the exploratory interaction analyses with lowered statistical restrictions (see Appendix 1 in Supplementary Material).
3. For the decisions in the aggressive provocative scenarios, the participants in the EG did not show any post-central activation patterns, whereas the NG participants did. This finding suggested that there was no expectation of physical pain in these rather adverse interactions in the EG (cf., Lamm et al., 2011), at least when they were presented in the fMRI scanner. In conclusion, in the EG, the aggressive provocative social interactions were processed quite differently from the NG. For instance, by not considering the possible negative consequences of their behavior, such as physical pain. An alternative explanation was that due to their socialization to violence, the EG did not form strong associations between aggressive behaviors and negative physical consequences (e.g., pain) in childhood and adolescence. The reason might be that in many social interactions, their aggressive and violent behaviors do not lead to negative consequences as the attacked person will not, or cannot fight back due to being hurt.
4. The aggressive provocative interaction scenarios produced a fusiform gyrus activation in the EG, but not in the NG. The fusiform gyrus seems to be related to many different functions, for example, object-representation (e.g., Haxby et al., 2001), the processing of faces (e.g., Kanwisher et al., 1997; Sabatinelli et al., 2011), and individual expert knowledge (e.g., Fehr, 2013). One can speculate that for the participants in the EG, an interpersonal threat might represent an area of expertise, which they feel like they will be able to handle due to their experiences with and socialization to violence. In accordance, the aggressive provocative interaction scenarios were rated as being more familiar in the EG than in the NG. To perceive an interpersonal threat as a highly familiar situation that can be managed, and that is not experienced as a kind of threat that should be avoided at any cost, might lead to dysfunctional associations between social conflicts and destructive aggressive behaviors. This association then strongly enhances the probability of inadequate aggressive behavior in social conflicts and might be learned in socialization shaped by above-average experiences of violence (cf., Cierpka et al., 2007; Fehr, 2012; Fehr et al., 2014).
  This argument was strengthened by the finding that the EG produced the activation of the fusiform gyrus only in the aggressive provocative interaction scenarios, whereas the NG generated this activation only in the social-positive interactions. Adding to the discussion above, one could argue that the individuals in the NG were *experts* in social-positive interactions, at least more so than being *experts* in aggressive-provocative interactions. This idea was further strengthened by the observation that the NG described the aggressive-provocative interaction scenarios as less familiar as the participants in the EG did. For this reason, the NG used their *expert* knowledge of how to respond to social-positive interaction scenarios, indicated by the activation of their fusiform gyri, while processing the social-positive interactions.
  Thus, the two groups of participants may be prone to recruit the same perceptual neural expert system in social behavior when assessing affective social interactions. However, the type of social interaction that activates this expert system, the social-positive or aggressive-provocative, is determined by the socialization to violence of the participants. For instance, the participants in the NG might have learned to be friendly in positive social interactions and define successful social interactions as highly sociable and presumably enjoyable ones. But, for the participants in the EG, success in social interactions might mean being the *winner* in terms of dominating other people, especially in aggressive provocative situations. This motivation results in aggressive behaviors in EG participants (cf., Blair, 2010, for an extensive discussion on neural correlates of reactive and instrumental aggression).
5. The involvement of the cerebellar brain regions in the EG while watching the aggressive-provocative interactions might point to a more basic over-learned and reflexive motor script activation (cf., Moulton et al., 2011). This motor script activation might facilitate physical violence in the EG participants under some circumstances. But the involvement of the thalamus adjacent to the caudate body, the putamen, the globus pallidus, and the claustrum in the NG, might reflect an activation of the motor system that could be located on a higher processing level affecting complex social decision making. Therefore, this motor system may be regulated more efficiently by the cognitive top-down control in the NG than in the EG (cf., Rosenbloom et al., 2012).

The results listed above, and their interpretation, might be useful to suggest some hypotheses about the possible socialization effects on the development of delinquent behavior. Neural resources, to be explored in future studies, might be recruited to control unwanted behavior when participating in rehabilitation programs to control aggressive behavior. These neural resources might become evident in the individual physiological data in the long run, i.e., after a couple of months when the participants successfully attend a rehabilitation program. Maybe observing such newly recruited neural resources as examined by the respective fMRI data could serve as one of the different indicators of the success of a rehabilitation program. Note that we do not argue that delinquent behaviors might have a genetic basis, instead, we claim that our data support the idea that the learning histories of individuals on the appropriateness of aggressive behaviors might substantially modulate violent behavior. In accordance with this idea, the heteromodal association cortices assumed to be recruited when encoding individual learning histories (cf., Fuster, 2006, 2009), play an important role in the present study. In line with this argument, Fehr et al. (2014) proposed the neural elaboration of the complex idiosyncratically learned perception-action cycles for the complex prototypic behaviors in the posterior (e.g., emotional body language system, see De Gelder, 2006) and frontal (e.g., action-programs) hetero-modal association cortices with violent behavior as a prominent example.

## Conclusion

Most of the brain regions involved in the response to aggressive-provocative interactions were recruited in both the delinquent and non-delinquent participants. But there were also qualitative (partially different activation patterns) and quantitative (percentage signal change values in several ROIs) differences in the brain activation between the two groups of participants when processing aggressive-provocative interactions. Note that between the two groups of participants, both similarities and differences in the recruited brain areas were particularly located across the heteromodal association cortices in the pre-and the post-central iso-cortex. These brain regions can be assumed to be characterized by high plasticity. Hence, these areas provide the ideal basis for learning processes (cf., Fuster, 2009). There is therefore good reason to hope that newly learned strategies of aggression control, for instance in resocialization programs, can reduce the probability of violent behaviors in the long run.

### Limitations of the Present Study

We included a notable sample of 30 non-student participants split into two, relatively small, but carefully selected, homogeneous, and matched sub-samples. The participants indicated their responses to the social interactions displayed in the video clips in an fMRI session. But they could not show open behaviors as they could in similar real-life situations. This, of course, limited the validity of the responses of the participants (i.e., their decision on how to respond to the respective interaction). Despite this restriction, we still believe that our experimental paradigm came close to real-life situations as far as what is possible in an fMRI scanner.

As a second limitation, we would like to mention that some of our results have to be handled with care as they were based on medium effect sizes. However, this is a general problem in the studies on complex mental phenomena (Lieberman and Cunningham, 2009; Fehr, 2013; Fehr and Milz, 2019). Therefore, and as our main hypotheses were based on previous studies on the topic, we think that our approach provides a valid and reliable basis for subsequent studies.

A third limitation came with the category of aggression that we focused on, which is reactive aggression. Other forms of aggressive behavior such as relational aggression, verbal aggression, aggression to defend attacked persons, and instrumental aggression (Blair, 2010) might produce different neural responses. Hence, future research should further develop the present approach to explore more peculiarities of emotional regulation networks involved in the control of different kinds of aggression.

## Data Availability Statement

The raw data supporting the conclusions of this article can be made available by the authors for local and offline examination. They will however not be sent via the internet for data protection reasons. Further inquiries can be directed to the corresponding author.

## Ethics Statement

The studies involving human participants were reviewed and approved by Geschäftsstelle der Ethikkommission, Universität Bremen, Bibliothekstr. 1, 28359 Bremen. The patients/participants provided their written informed consent to participate in this study.

## Author Contributions

JW: conceptualization, formal analysis, investigation, writing—original draft, and visualization. AJ: resources, project administration, investigation, and writing—review and editing. AA: writing—review and editing and supervision. TF: conceptualization, methodology, software, visualization, investigation, writing—original draft, supervision, and project administration. All authors contributed to the article and approved the submitted version.

## Conflict of Interest

The authors declare that the research was conducted in the absence of any commercial or financial relationships that could be construed as a potential conflict of interest.

## Publisher's Note

All claims expressed in this article are solely those of the authors and do not necessarily represent those of their affiliated organizations, or those of the publisher, the editors and the reviewers. Any product that may be evaluated in this article, or claim that may be made by its manufacturer, is not guaranteed or endorsed by the publisher.
