# Supplementary material for: Neural Correlates of Decisions in Quasi-Realistic, Affective Social Interactions in Individuals With Violence-Related Socialization
Source: Front Behav Neurosci. 2021 Oct 21;15:713311. doi: 10.3389/fnbeh.2021.713311 (PMC8566670; doi:10.3389/fnbeh.2021.713311)
Supplement: Supplementary file 1 [file Table_1.DOC]

# Supplementary online material

The following appendix is meant to inspire future research on the topic by additional interaction analyses as basis for further hypotheses building.

Appendix 1 – Interaction analyses

| **Interaction analyses on contrast aggressive provocative vs. neutral interaction scenarios: EG > NG, NG > EG** | | | | | | | | | |
| --- | --- | --- | --- | --- | --- | --- | --- | --- | --- |
|  |  | **EG > NG** | | | | **NG > EG** | | | |
| **anatomical region** | **H** | **t** | **x** | **y** | **z** | **t** | **x** | **y** | **z** |
|  |  |  |  |  |  |  |  |  |  |
| Inferior Frontal Gyrus | L | 2.3 | -32 | 32 | 15 | 2.2 | -36 | 7 | 24 |
|  | R | 2.2 | 50 | 1 | 24 | 2.7 | 50 | 19 | -6 |
|  | R | 2 | 48 | 43 | 13 | 2.2 | 57 | 12 | 10 |
| between Cingulate Gyrus and Paracentral Lobule | R |  |  |  |  | 2 | 0 | -27 | 42 |
| Anterior Cingulate | L |  |  |  |  | 2.9 | -14 | 43 | 7 |
|  | R |  |  |  |  | 3 | 8 | 35 | 4 |
|  | R |  |  |  |  | 2.5 | 12 | 41 | -4 |
|  | R |  |  |  |  | 2 | 2 | 22 | 12 |
| Cingulate Gyrus | L | 2.2 | -10 | -10 | 43 | 2.5 | -14 | 18 | 40 |
|  | L |  |  |  |  | 2.2 | -10 | -4 | 32 |
|  | L |  |  |  |  | 2.1 | -10 | 4 | 40 |
|  | R | 2.1 | 10 | 6 | 35 | 2.6 | 10 | -37 | 31 |
|  | R | 2 | 4 | -6 | 43 |  |  |  |  |
| Posterior Cingulate | L |  |  |  |  | 1.9 | 0 | -30 | 20 |
| between Insula and Cingulate Gyrus | R |  |  |  |  | 2.6 | 26 | -40 | 24 |
| Insula | R |  |  |  |  | 3.2 | 48 | 10 | 5 |
|  | R |  |  |  |  | 2.3 | 36 | -15 | 10 |
|  | R |  |  |  |  | 2.3 | 42 | -21 | 14 |
| Parahippocampal Gyrus | L |  |  |  |  | 3.6 | -38 | -24 | -17 |
|  | L |  |  |  |  | 1.9 | -18 | -39 | 0 |
|  | R | 2 | 38 | -51 | -1 | 3.2 | 14 | -9 | -18 |
|  | R |  |  |  |  | 2.5 | 36 | -35 | -7 |
|  | R |  |  |  |  | 2.1 | 24 | -32 | -12 |
| between MTG, STG, HIP ,and Parahippocampal G. | R |  |  |  |  | 2.1 | 32 | -46 | 8 |
| Amygdala | R |  |  |  |  | 2.5 | 20 | -3 | -12 |
| Hippocampus | R |  |  |  |  | 2.6 | 30 | -37 | -2 |
| Thalamus | L |  |  |  |  | 2.8 | -18 | -23 | 3 |
|  | R |  |  |  |  | 2.3 | 16 | -9 | 6 |
| Midbrain | L |  |  |  |  | 2.2 | -2 | -26 | -16 |
|  | R | 2.4 | 12 | -19 | -1 | 2.8 | 6 | -30 | -9 |
| Pons | L |  |  |  |  | 2.2 | -18 | -19 | -28 |
|  | L |  |  |  |  | 2.2 | -6 | -38 | -27 |
|  | L |  |  |  |  | 2.1 | -2 | -19 | -31 |
|  | L |  |  |  |  | 1.9 | -10 | -23 | -27 |

**Tab. S1:** Anatomical regions, peak activation t-values, and Talairach-coordinates for exploratory interaction analyses (including inferior frontal, limbic, and sub-cortical brain regions) between EG and NG based on the contrast aggressive provocative vs. neutral interaction scenarios; H = hemisphere: L = left, R = right, all statistics p < .05, uncorrected, minimum voxel cluster size k = 5 voxels.
